# AAV‐mediated Gene Cocktails Enhance Supporting Cell Reprogramming and Hair Cell Regeneration

**DOI:** 10.1002/advs.202304551

**Published:** 2024-05-29

**Authors:** Liyan Zhang, Xin Chen, Xinlin Wang, Yinyi Zhou, Yuan Fang, Xingliang Gu, Ziyu Zhang, Qiuhan Sun, Nianci Li, Lei Xu, Fangzhi Tan, Renjie Chai, Jieyu Qi

**Affiliations:** ^1^ State Key Laboratory of Digital Medical Engineering Department of Otolaryngology‐Head and Neck Surgery Zhongda Hospital School of Life Sciences and Technology School of Medicine Advanced Institute for Life and Health Jiangsu Province High‐Tech Key Laboratory for Bio‐Medical Research Southeast University Nanjing 210096 China; ^2^ Department of Otolaryngology‐Head and Neck Surgery Shandong Provincial ENT Hospital Shandong University Jinan 250022 China; ^3^ Shandong Institute of Otorhinolaryngology Jinan 250022 China; ^4^ Co‐Innovation Center of Neuroregeneration Nantong University Nantong 226001 China; ^5^ Department of Neurology Aerospace Center Hospital School of Life Science Beijing Institute of Technology Beijing 100081 China; ^6^ Department of Otolaryngology‐Head and Neck Surgery Sichuan Provincial People's Hospital School of Medicine University of Electronic Science and Technology of China Chengdu 610072 China; ^7^ Southeast University Shenzhen Research Institute Shenzhen 518063 China

**Keywords:** AAV, hair cell regeneration, multi‐gene co‐regulation

## Abstract

Mammalian cochlear hair cells (HCs) are essential for hearing, and damage to HCs results in severe hearing impairment. Damaged HCs can be regenerated by neighboring supporting cells (SCs), thus the functional regeneration of HCs is the main goal for the restoration of auditory function in vivo. Here, cochlear SC trans‐differentiation into outer and inner HC by the induced expression of the key transcription factors Atoh1 and its co‐regulators Gfi1, Pou4f3, and Six1 (GPAS), which are necessary for SCs that are destined for HC development and maturation via the AAV‐ie targeting the inner ear stem cells are successfully achieved. Single‐cell nuclear sequencing and lineaging tracing results showed that the majority of new Atoh1‐derived HCs are in a state of initiating differentiation, while GP (Gfi1, Pou4f3) and GPS (Gfi1, Pou4f3, and Six1) enhanced the Atoh1‐induced new HCs into inner and outer HCs. Moreover, the patch‐clamp analysis indicated that newborn inner HCs induced by GPAS forced expression have similar electrophysiological characteristics to those of native inner HCs. Also, GPAS can induce HC regeneration in the HC‐damaged mice model. In summary, the study demonstrates that AAV‐mediated co‐regulation of multiple genes, such as GPAS, is an effective means to achieve functional HC regeneration in the mouse cochlea.

## Graphical abstract

AAV‐ie mediated Atoh1/GPA/GPAS overexpression can regenerate HC‐like cells. Most Atoh1‐induced HC‐like cells are in a state of initiating differentiation, while GP and GPS enhance the differentiation of HC‐like cells induced by Atoh1 into IHC‐like cells and OHC‐like cells. And, GPAS‐induced IHC‐like cells have similar electrophysiological characteristics to the native IHCs. In addition, GPAS can induce HC‐like cell regeneration in a mouse model with AAV‐DTR‐induced HC damage.

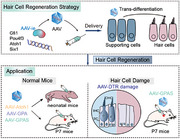

## 1 Introduction

Auditory sensory hair cells (HCs) and surrounding nonsensory supporting cells (SCs) differentiate from the common pool of inner ear progenitor cells. HCs in the sensory epithelium are precisely arranged into outer HCs (OHCs) and inner HCs (IHCs), intermixed with various types of SCs, including Deiter’ cells (DCs), Hensen's cells, inner border cells (IBCs), and inner phalangeal cells (IPhCs), etc.^[^ ^1^ ^]^ Sensory HCs act as the mechanoreceptors that transform acoustic waves into electrical signals transmitted to the brain through spiral ganglion neurons innervating the HCs. As terminally differentiated cells, mammalian cochlear HCs are susceptible to genetic mutations and environmental stress. Irreversible damage to HCs is one of the leading causes of sensorineural hearing loss in mammalian or mammalian species.^[^ ^2^, ^3^ ^]^

Some SCs in the inner ear have the ability to proliferate and to be reprogrammed to differentiate into HCs, and these are known as sensory progenitors and are distributed in the lesser epithelial ridge, the greater epithelial ridge (GER), the vestibular sensory epithelium, and the cristae of the semicircular canals.^[^ ^4^ ^]^ New HCs are obtained through miotic HC regeneration and/or direct SC‐to‐HC trans‐differentiation.^[^ ^5^ ^]^ Promoting functional HC regeneration by co‐regulating signaling pathways to activate sensory precursors and induce HC differentiation represents a promising therapeutic strategy for sensorineural hearing loss.

SCs proliferation and differentiation into HCs are regulated by multiple signaling pathways.^[^ ^6^ ^]^ Key transcription factors include Atoh1, Sox2, Gfi1, Six1, Pou4f3, etc. in the development of cochlear epithelia, and the interplay between these regulators is responsible for gene expression and fate determination. As a transcription‐activating gene belonging to the basic helix‐loop‐helix family, Atoh1 plays a crucial role as a positive regulator in directing the differentiation of cochlear cells in mammals.^[^ ^7^ ^]^ Precise regulation of Atoh1 during HC genesis and development is achieved through upstream and downstream regulatory mechanisms to ensure developmental precision.

The transcription factors Sox2, Six1, and Eya1 cooperate to initiate Atoh1 expression through direct binding of Sox2 and Six1 to the Atoh1 3′ enhancer.^[^ ^8^ ^]^ Overexpression of Atoh1 leads to the ectopic formation of HCs in the cochlea. Despite many promising reports, there are significant variations in regeneration capacity and mature function of HCs upon overexpression of Atoh1 in mice,^[^ ^9^, ^10^, ^11^ ^]^ with the regeneration efficiency declining sharply with age.^[^ ^12^ ^]^ Therefore, Atoh1 alone is insufficient for regenerating mature HCs with physiological functions, necessitating the involvement of additional transcription factors for functional regeneration.

The POU domain transcription factor Pou4f3, a downstream target gene of Atoh1, plays an essential role in the differentiation and survival of mature HC.^[^ ^13^ ^]^ Pou4f3 promotes Atoh1‐mediated HC development and survival by regulating the expression of Gfi1 and Nr2f2, which are downstream target genes related to HC regeneration.^[^ ^14^, ^15^, ^16^ ^]^ Previous studies have shown that co‐expression of some factors (Gfi1, Pou4f3, and Atoh1 (GPA)) is more effective than overexpression of Atoh1 alone in inducing transformation of embryonic stem cells into HC‐like cells in vitro,^[^ ^17^ ^]^ and the same conclusion applies to the cochlea where combined regulation of GPA enhances trans‐differentiation from SC to HC.^[^ ^18^, ^19^, ^20^ ^]^ The joint regulation of GPA can promote the further development and maturation of regenerated HCs to a certain extent, as evidenced by increased activity levels of Slc26a5 and Vglut3. However, the electrophysiological function of the newly regenerated HCs still cannot achieve a level of maturity comparable to in situ HCs and lacks mature mechanical responses.^[^ ^18^ ^]^

In 2020, the study showed a combination of 4 transcription factors (GPAS, Gfi1, Pou4f3, Atoh1 and Six1) can reprogram neonatal mouse SCs (postnatal day (P)8), adult mouse tail fibroblasts, and mouse embryonic fibroblasts into HC‐like cells in vitro.^[^ ^21^ ^]^ These HC‐like cells possess various classical characteristics of HCs, including morphological, epigenetic, and transcriptomic features, electrophysiological features, mechanosensory channels, and ototoxic sensitivity. And, in 2024, the paper proved GPAS‐induced HC regeneration in the flat epithelium of mature guinea pigs.^[^ ^22^ ^]^ *Six1* is an upstream gene of *Atoh1* that regulates the expression of Atoh1 via Eya1 and Sox2.^[^ ^8^ ^]^ Six1 directly targets the Atoh1 auto‐regulatory enhancer and regulates a series of downstream factors including Pou4f3, Gfi1, Gata3, and Pbx1.^[^ ^23^ ^]^ Moreover, the previous study has shown that Six1 plays a role in HC maturation by regulating pivotal genes involved in establishing hair bundle orientation and planar cell polarity such as *Cdc42*, *Myo6*, and *Vangl2* etc.^[^ ^23^ ^]^ Therefore, as an upstream regulatory transcription factor of key genes involved in various stages of HC development and maturation, Six1 is likely to play a positive functional role in the regeneration of endogenous functional HCs in the cochlea.

The aforementioned evidence suggests that the coordinated regulation of multiple genes is an effective strategy for achieving functional HC regeneration in vivo. However, achieving such coordination in the mouse cochlea poses challenges. Most of the traditional methods involve constructing transgenic mice and using the Cre (CreER)/loxp system to regulate gene regulation in the inner ear stem cells. However, this has limitations, for example, a long experiment period in the application of multi‐gene regulation, to which AAV‐mediated polygene regulation is the perfect solution. We previously identified a novel and safe AAV variant, AAV‐ie,^[^ ^24^ ^]^ which has demonstrated exceptional efficiency at infecting inner ear stem cells. Therefore, here we employed AAV‐ie to overexpress key genes, including *Atoh1*, GPA, and GPAS, in the SCs of the juvenile mouse cochlea to achieve massive regeneration of efficient OHC‐like cells and IHC‐like cells. Meanwhile, relative to Atoh1, GPA and GPAS could simultaneously achieve regeneration of a large number of relative mature OHC‐like cells and IHC‐like cells in vivo, and relative mature OHC‐like cells were derived from the Lgr5^+^ DCs lateral to the IHCs. Single‐cell nuclear sequencing showed that these GPAS‐generated HC‐like cells were significantly more advanced than the HCs obtained in earlier differentiation experiments. Notably, single‐cell trajectory analyses showed that when Atoh1 alone led to the generation of immature HC‐like cells, while GPA/GPAS‐induced nascent HC‐like cells developed more temporally and approached the state of in situ OHCs and IHCs. The sequencing results were confirmed by lineage tracing of HC‐like cells. Furthermore, electrophysiological experiments demonstrated that the regenerated HC‐like cells adjacent to the IHC region in the GPAS injected group displayed the same electrophysiological characteristics as native HCs in mice of the same age. In addition, GPAS can induce HC‐like cell regeneration in a mouse model with AAV‐DTR (diphtheria toxin receptor)‐induced HC damage. Our experimental findings thus propose an efficient approach to achieving robust and efficient production of mature HC‐like cells in vivo and suggest that AAV‐mediated GP (Gfi1,Pou4f3) and GPS (Gfi1, Pou4f3 and Six1) could serve as additional therapeutic targets for Atoh1‐mediated HC regeneration and maturation.

## 2 Results

### 2.1 AAV‐*Atoh1*, AAV‐GPA, and AAV‐GPAS Successfully Induced the Regeneration of HC‐like Cells In Vivo

**Figure 1:**
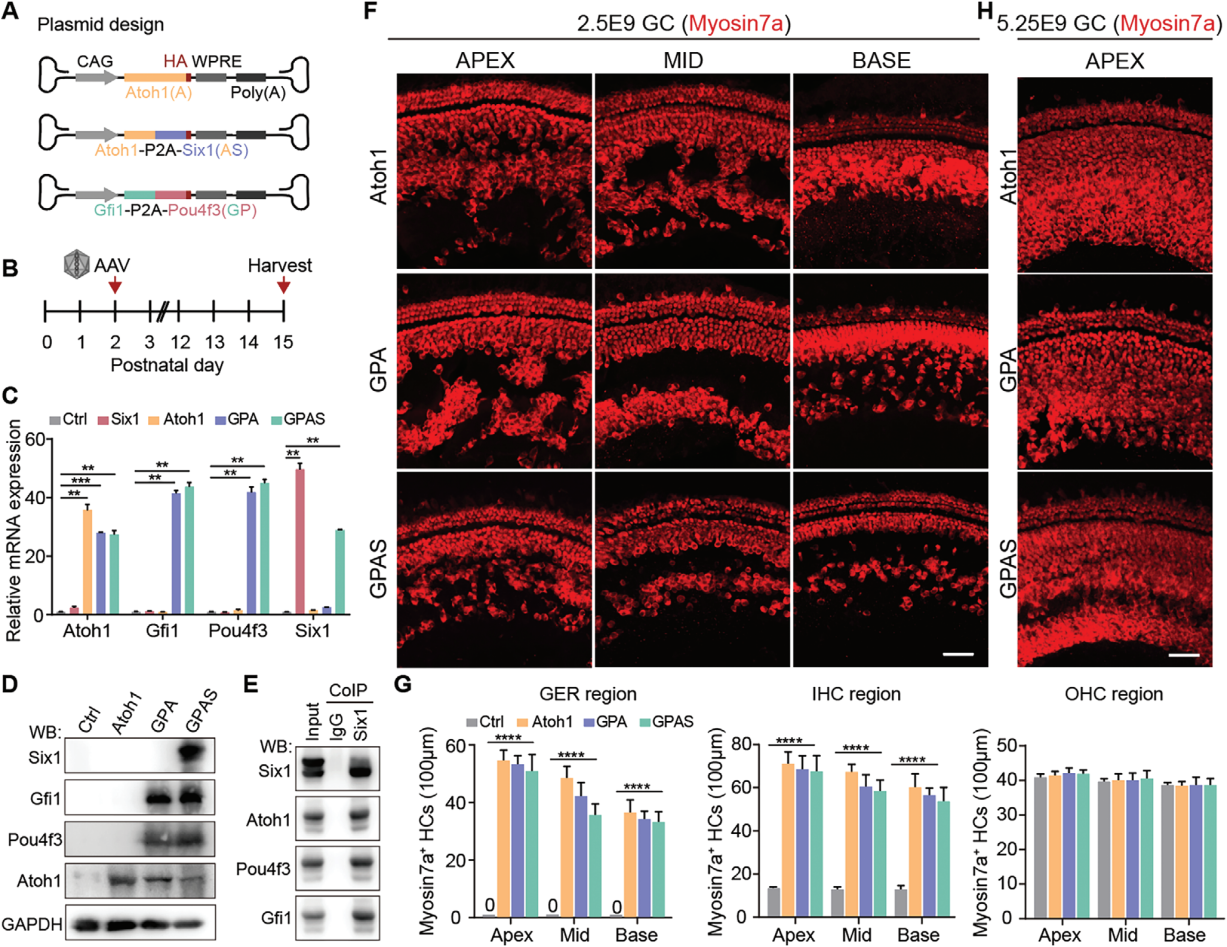
AAV‐ie mediated Atoh1, GPA, and GPAS effectively regenerated HC‐like cells in vivo. A) Strategy for the overexpression of exogenous Atoh1 (A), Atoh1‐Six1 (AS), and Gfi‐Pou4f3 (GP) mediated by AAV‐ie in the neonatal mouse inner ear. The P2A polypeptide sequence mediates protein self‐splicing after translation. All the proteins were tagged with an HA (hemagglutinin) tag. B) Experimental design diagram. All the AAVs were delivered to the mice's left ear at dose:2.5E9 GCs per ear, then the cochleae were obtained at P15. C) The qPCR showing the relative mRNA expression of *Atoh1*, *Gfi1*, *Pou4f3*, and *Six1* in the cochleae injected with AAV‐control, AAV‐*Atoh1*, AAV‐*Six1*, AAV‐GPA, and AAV‐GPAS. Raw results from 3 replicated experiments. Error bars are ±SEM. ^**^ *p* < 0.01, ^***^ *p* < 0.001. D) Representative immunoblotting images of Atoh1, Gfi1, Pou4f3, and Six1 in the cochleae injected with AAV‐control, AAV‐*Atoh1*, AAV‐GPA, and AAV‐GPAS. 4 repeated experiments, and 1 representative images. E) The western blotting images of Atoh1, Gfi1, Pou4f3, and Six1 in the left ear were injected with AAV‐GPAS. Six1 antibody was used for the immunoprecipitation, and western blotting was used to analyze the co‐expressed proteins using Atoh1, Pou4f3, or Gfi1 antibody. 4 repeated experiments, and 1 representative image. F) Representative confocal images of Myosin7a (red, HC marker) signals in the basal, middle, and apical turns of the ear injected with AAV‐*Atoh1*, AAV‐GPA, and AAV‐GPAS. All the AAVs were delivered to the mice's left ear at a dose of 2.5E9 GCs per ear. Scale bar, 50 µm. G) The number of Myosin7a in the AAV‐*Atoh1*, AAV‐GPA, and AAV‐GPAS injected cochlea in (F). *N* = 5, error bars are ±SEM, ^****^ *p* < 0.0001. H) Representative confocal images of Myosin7a (red, HC marker) signals in the apical turns of the ear injected with AAV‐*Atoh1*, AAV‐GPA, and AAV‐GPAS. All the AAVs were delivered to the mice's left ear, at a dose of 5.25E9 GCs per ear. Scale bar, 50 µm.

The transgene capacity for AAV packaging is limited to 4.7 kb,^[^ ^25^, ^26^ ^]^ and the coding region length of *Gfi1*, *Pou4f3*, *Atoh1*, and *Six1* is 1272, 1017, 855, and 1056 bp, respectively. In order to achieve the co‐regulation of GPA and GPAS, we constructed vectors containing *Atoh1*, *Atoh1*‐P2A‐Six1 (AS), and *Gfi1*‐P2A‐*Pou4f3* (GP) for AAVs, respectively (**Figure** **1**A). AAV‐ie was used as the capsid protein for virus packaging. A previous report showed that AAV‐ie had very high efficiency in HCs and SCs, and here we examined the efficiency in postnatal mice (Figure S1, Supporting Information). And AAV‐ie‐mediated 2 AAVs have high efficiency in the same cells (Figure S2, Supporting Information). Therefore, the viruses were injected into P2 wild‐type mice through the round window membrane (RWM) (Figure 1B). The expression of these 4 exogenous genes at the transcriptome level and protein level were measured, and the quantitative real‐time PCR (qPCR) results and the western blotting results verified the overexpression of Atoh1, GPA, and GPAS in corresponding cochleae. After AAV virus injection, the expression of all 4 genes increased dramatically (Figure 1C,D), indicating the successful expression of AAV‐ie‐mediated gene transfer into the cochlea. Previous reports showed that *Six1* is the upstream gene of *Atoh1*, *Gfi1*, and *Pou4f3*,^[^ ^23^ ^]^ and we also verified this result by the co‐immunoprecipitation experiment in the AAVs injected mouse cochlea (Figure 1E).

Next, immunofluorescence staining of Myosin7a, a marker for HCs, was performed at P15, and we found new HC‐like cells induced by *Atoh1*, GPA, and GPAS overexpression in the region of OHCs, IHCs, and the GER (Figure 1F,G). Also, increasing the number of genome‐containing particles (GCs) of AAV‐*Atoh1*, AAV‐GPA, and AAV‐GPAS could promote greater ectopic HC‐like cell regeneration in the cochlea compared with the low GC groups (Figure 1F,H).

We also observed the effect of *Six1* alone on HC regeneration. And the imaging data showed that there were no newly formed HC‐like cells in AAV‐*Six1* transduce cochlea (Figure S3, Supporting Information). In addition, we found that almost all newly regenerated ectopic HC‐like cells by *Atoh1* up‐regulation expressed Sox2, an SC marker, indicating that the newly formed HC‐like cells were derived from SCs (Figure S4, Supporting Information). Strong Sox2 signals in Myosin7a^+^ cells mean that the regenerated HC‐like cells were in the early stages of trans‐differentiation from SCs and thus were immature. The administration of AAV‐GP decreased the expression of Sox2 in AAV‐*Atoh1*‐induced newly regenerated HC‐like cells, and this decline in Sox2 expression was enhanced by AAV‐*Six1* (Figure S4, Supporting Information), indicating that GPS overexpression enabled the regenerated HC‐like cells to reach a relatively mature state.

### 2.2 Single‐Nucleus Transcriptomic Analysis Revealed the Level of Maturity in Newly Regenerated HC‐like Cells

**Figure 2:**
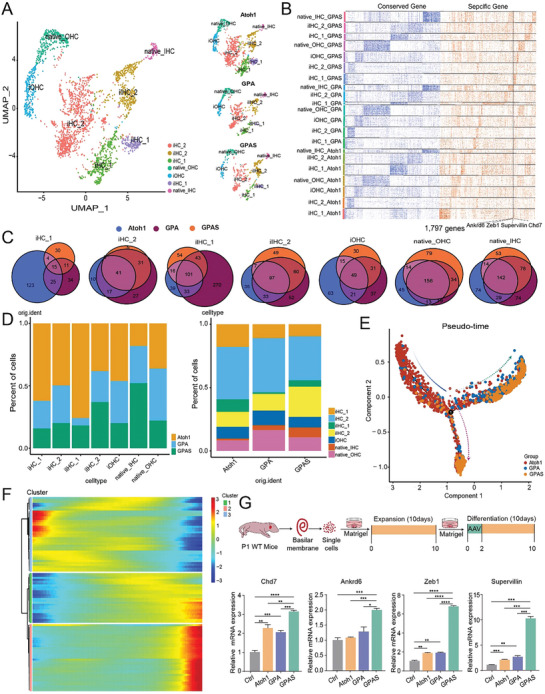
Single‐nucleus transcriptomic analysis and cell trajectory analysis of AAV‐induced HCs. A) UMAP plot showing seven potential subtypes HCs in the AAV‐*Atoh1*, AAV‐GPA, and AAV‐GPAS groups. B) Heatmap illustrating shared and group‐specific differentially expressed genes in each subtype related to (A). C) Venn diagrams showing differentially expressed genes in each subtype from the AAV‐*Atoh1* (blue) and AAV‐GPA (violet) groups compared to the AAV‐GPAS group (orange). D) The proportions of HC subtypes from the 3 groups using the Seurat R package. E) The trajectory manifold of AAV‐infected HCs from the AAV‐*Atoh1*, AAV‐GPA, and AAV‐GPAS groups using the Monocle 2 algorithm. Dotted and solid lines show the different cell fates /trajectories confirmed by expression profiles. F) Heatmap of significant genes according to branch expression analysis comparing the HC fates in the 3 groups. The genes in the heatmap were used to analyze the pseudotime‐based variation. G) Top: Illustration of the experimental design. AAVs were added to the culture medium, dose: 3E10 GCs per well. Bottom: The qPCR data showed the transcription‐level expression of *Chd7*, *Ankrd6*, *Zeb1*, and *Supervillin* mRNA in the AAV‐Ctrl/Atoh1/GPA/GPAS overexpressing organoids. Raw results from 3 replicated experiments. Error bars are ±SEM. ^*^ *p < *0.05, ^**^ *p < *0.01, ^***^ *p* < 0.001, ^****^ *p* < 0.0001.

Next, we asked whether co‐regulation with a combination of transcription factors could increase HC regeneration and their maturity to closely mimic the native HCs in vivo. We profiled the single‐nucleus transcriptomes from cochlear epithelium regions conditioned with the AAV‐*Atoh1*, AAV‐GPA, or AAV‐GPAS groups. After initial quality control and doublet removal, we succeeded in capturing a total of 2722 single nuclei from the 3 AAV‐injected groups, and then an unbiased clustering analysis was used to identify cochlear cell types. Using classical HC markers, including *Tmc1*, *Slc26a5*, *Otof*, and *Atoh1*, we clearly identified a small proportion of HC types that also included AAV‐injected cells because all AAV‐injected populations overexpressed *Atoh1*. We further took subsets of these potential HC types from each group and re‐clustered them to obtain AAV‐transfected HC‐like cells and native HCs, respectively (Figures S5–S7, Supporting Information). We merged 4 HC subpopulations from each group by using joint CCA‐embedding. Most of the *Atoh1* ^+^ cells were regarded as regenerated HCs. *Tmc1*, *Slc26a5*, and *Otof* were the markers of HCs, OHCs, and IHCs, respectively. Thus, using a combination of similar gene expression patterns in each cluster from each group and pseudotime‐based expression patterns of *Otof*, *Slc26a5*, *Tmc1*, and *Atoh1*. Then we could manually annotate 7 subtypes including a native OHC cluster and a native IHC cluster, 2 induced HC clusters (iHC 1 and 2), an induced OHC cluster (iOHC), and 2 induced IHC clusters (iIHC 1 and 2) (**Figure** **2**A; Figure S8, Supporting Information).

Next, we analyzed the differential gene expression in the AAV‐*Atoh1*, AAV‐GPA, and AAV‐GPAS transduced cochlea (Figure 2B,C; Figure S9, Supporting Information). Previous reports have shown that *Pbx1* is a functional *Six1* target gene that plays a similar role in the feedforward regulation of cochlear sensory epithelial development.^[^ ^23^ ^]^ In our sequencing data, *Pbx1* was highly expressed in the AAV‐GPAS group compared to the AAV‐*Atoh1* and AAV‐GPA groups. Similarly, other HCs development and maturation genes including *Supervillin*,^[^ ^27^ ^]^ *Zeb1*,^[^ ^28^, ^29^ ^]^ *Chd7*,^[^ ^30^ ^]^ and *Ankrd6* ^[^ ^31^ ^]^ were highly expressed in the AAV‐GPAS group. We compared the HC composition of cochlear samples injected with AAV‐*Atoh1*, AAV‐GPA, and AAV‐GPAS (Figure 2D). The proportion of iHCs, iOHCs, and iIHCs in the AAV‐*Atoh1*, AAV‐GPA, and AAV‐GPAS groups was high, indicating that Atoh1 successfully induced HC regeneration. The proportion of native OHCs and iOHCs in the AAV‐GPA group was the highest, accounting for about 27%, which was slightly higher than in the other 2 groups, indicating that GP seemed to enhance the differentiation of OHCs. In contrast, the proportion of native IHCs and iIHCs in the AAV‐GPAS group was the highest, indicating that GPAS seemed to induce the differentiation of IHCs even though in the AAV‐GPA group the percentage of native IHCs and iIHCs was significantly higher than the AAV‐*Atoh1* group (Figure 2D). These data suggest that *Six1* might be essential for SC trans‐differentiation into IHCs. Therefore, to further distinguish iHCs from in situ HCs, OHCs, and IHCs (Figure S8E, Supporting Information), we tested the gene expression patterns during HC trans‐differentiation and development through Monocl2 trajectory analysis. We found that a large number of HC‐like cells in the AAV‐*Atoh1* group were at the origin of the pseudotime‐based trajectory, suggesting that more HC‐like cells were in an immature state. However, the cells of the AAV‐GPA and AAV‐GPAS groups showed branched trajectories, and the AAV‐GPA and AAV‐GPAS groups were densely distributed in both IHC and OHC trajectories. The AAV‐GPA group had more cells on the differentiation track of the OHC lineage, while the AAV‐GPAS group had more cells at the end of the development track of IHCs (Figure 2E). We further analyzed the gene expression patterns of all genes along the progression track of iHCs, and we further clustered these genes into 3 distinct expression modules (Cluster 1–3) (Figure 2F). The cochlear epithelia included different types of cells, and we used pure organoids derived from SCs alone to verify the sequencing results in vitro by qPCR. The qPCR results showed that *Supervillin*, *Zeb1*, *Chd7*, and *Ankrd6* were highly expressed in the AAV‐GPAS overexpressing organoids compared to the AAV‐*Atoh1* and AAV‐GPA organoids (Figure 2G), which was consistent with the sequencing data. Taken together, these data indicated that AAV‐GP/GPS induced more maturate HC‐like cells in AAV‐*Atoh1* induced newly regenerated HC‐like cells.

### 2.3 AAV‐GPA and AAV‐GPAS Enhanced the Trans‐Differentiation of SCs into OHC/IHC‐like Cells In Vivo

**Figure 3:**
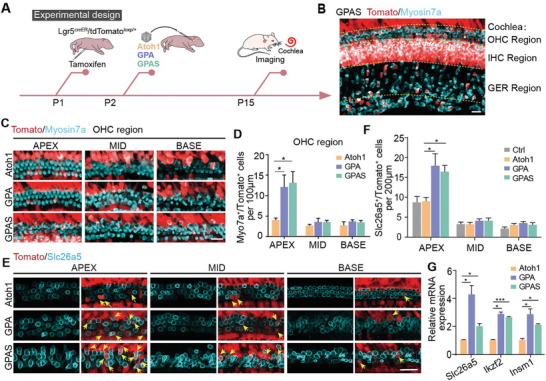
AAV‐ie‐mediated GP and GPS overexpression enhanced the OHC‐like cell regeneration. A) Experimental design diagram. All the AAVs were delivered to the mice's left ear, at dose:2.5E9 GCs per ear, then the cochleae were obtained at P15. B) Representative confocal images of Myosin7a and Tomato signals in the apical turns of the ear injected with AAV‐GPAS. Red: Tomato, cyan: Myosin7a. Scale bars, 20 µm. C) The immunofluorescence images of Myosin7a and Tomato signals in the basal, middle, and apical turns of the ear injected with AAV‐*Atoh1*, AAV‐GPA, and AAV‐GPAS. Magnified areas from the OHC region, Red: Tomato, cyan: Myosin7a. Scale bars, 20 µm. D) The number of Myosin7a (Myo7a)^+^/Tomato^+^ cells in the ear transduced by AAV‐*Atoh1*, AAV‐GPA, and AAV‐GPAS, corresponding to (C). *N* = 8, error bars are ±SEM. ^*^ *p < *0.05. E) Representative confocal images of Slc26a5 (an OHC marker) and Tomato signals in the basal, middle, and apical turns of the ear injected with AAV‐*Atoh1*, AAV‐GPA, and AAV‐GPAS. Arrows point to Slc26a5^+^/ Tomato^+^ regenerated HCs. Red: Tomato, cyan: Slc26a5. Scale bars, 20 µm. F) The number of double‐positive Slc26a5^+^/Tomato^+^ cells in the ear transduced by AAV‐*Atoh1*, AAV‐GPA, and AAV‐GPAS correspond to (E). *N* = 8, error bars are ±SEM. ^*^ *p < *0.05. (G) The qPCR showing the *Slc26a5*, *Ikzf2*, and *Insm1* mRNA expression in the ear transduced with AAV‐*Atoh1*, AAV‐GPA, and AAV‐GPAS, respectively. Raw results from 3 replicated experiments. Error bars are ±SEM. ^*^ *p* < 0.05, ^***^ *p* < 0.001.

Mammalian cochlear SCs act as progenitors for HC regeneration, and these SCs include at least eight subtypes, namely DCs, Hensen's cells, IPCs (inner pillar cells), OPCs (outer pillar cells), IBCs, IPhCs, Claudius cells, and the cells in the GER region.^[^ ^32^, ^33^ ^]^ AAV‐ie has been shown to have high transduction efficiencies in DCs, OPCs, IPCs, and IBC/IPhCs at ≈75%, 90%, 80%, and 90%, respectively.^[^ ^24^ ^]^ Our previous report showed that Lgr5^+^ DCs, IPCs, and IPhCs/IBCs were the main sources of regenerated HCs.^[^ ^34^, ^35^ ^]^ Thus, Lgr5^CreER^/tdTomato^loxp/+^ mice were used to trace the regenerated HCs. The Cre enzyme was activated by injecting tamoxifen intraperitoneally in the Lgr5^CreER^/tdTomato^loxp/+^ mice at P1, next corresponding AAVs were injected into the ear through the RWM at P2. Cochlear epithelia were collected 2 weeks later for immunofluorescence staining (**Figure** **3**A). Myosin7a^+^/Tomato^+^ HC‐like cells were detected in AAV‐GPAS injected cochleae, including the OHC region, IHC region, and GER region (Figure 3B). A previous report showed that *Pou4f3* and *Gfi1* promote the capacity of Atoh1‐mediated HC generation in the mice,^[^ ^18^ ^]^ and we verified this result using AAV‐GPA and AAV‐GPAS (Figure 3C). Also, we found that the number of Myosin7a^+^/Tomato^+^ HC‐like cells was greater in the AAV‐GPA and AAV‐GPAS transduced cochleae compared with AAV‐*Atoh1* (Figure 3D).

OHCs are responsible for amplifying the mechanical vibration of perceived sound signals, and this activity depends on the motor protein Slc26a5, which is especially expressed in the lateral wall of OHCs.^[^ ^36^ ^]^ Therefore, Slc26a5 is considered a functional molecular marker of mature OHCs. We traced new HC‐like cells in the cochleae from Lgr5^CreER^/tdTomato^loxp/+^ mice injected with AAV‐*Atoh1*, AAV‐GPA, and AAV‐GPAS (Figure 3E), and we defined all OHCs derived from DCs as Tomato^+^/ Slc26a5^+^ double‐positive cells. New HCs generated from Atoh1 ectopic expression in cochlear progenitors do not express Slc26a5,^[^ ^9^, ^37^ ^]^ and this phenomenon was verified in the AAV‐*Atoh1*‐transduced cochlea (Figure 3F). In order to further improve the maturity of new OHCs from DCs, we introduced Gfi1, Pou4f3, and Six1 similar to AAV‐*Atoh1*. Compared with the overexpression of Atoh1 alone, more newly formed OHCs derived from DC trans‐differentiation (Tomato^+^/ Slc26a5^+^) were detected in response to AAV‐GP or AAV‐GPS enhancement (Figure 3E,F). Also, the qPCR results showed that the transcriptome expression levels of *Ikzf2* ^[^ ^38^ ^]^ and *Insm1*,^[^ ^39^ ^]^ which determine OHC development, were higher in the GPA group (Figure 3G). Compared with the 3 groups, the GPA‐transduced cochlear epithelium showed more Tomato^+^/ Slc26a5^+^ OHC‐like cells and greater Slc26a5 expression at the transcription level (Figure 3E–G). These results indicated that AAV‐GP and GPS could promote the formation and functional maturation of new OHC‐like cells, which is consistent with the results in AAV‐GP and GPS overexpressing mice and single‐cell nuclear sequencing data.

**Figure 4:**
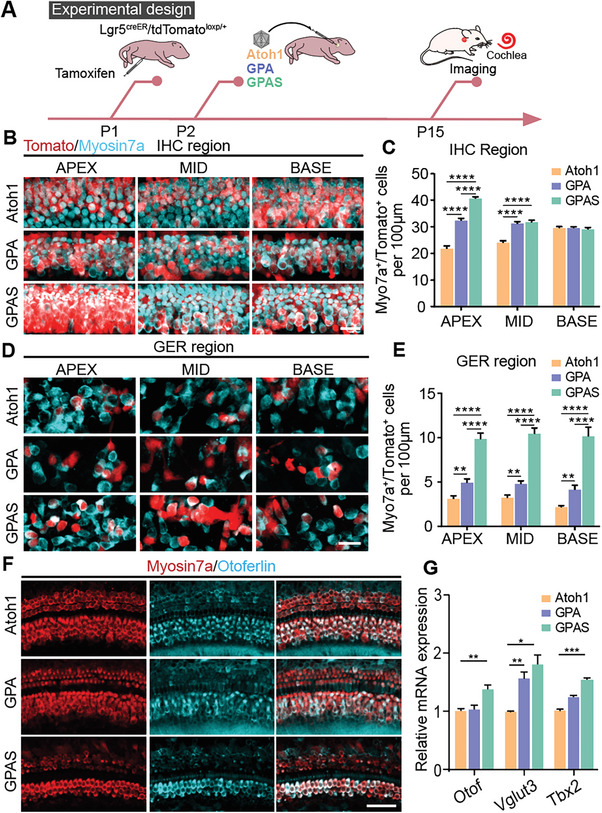
AAV‐ie‐mediated GPS overexpression strongly promoted the efficiency of *Atoh1*‐indued HC‐like cell regeneration in the IHC region. A) Experimental design diagram. All the AAVs were delivered to the mice's left ear, at dose:2.5E9 GCs per ear, then the cochleae were obtained at P15. B) The immunofluorescence images of Myosin7a and Tomato signals in the basal, middle, and apical turns of the ear injected with AAV‐*Atoh1*, AAV‐GPA, and AAV‐GPAS. Magnified areas from the IHC region corresponding to (Figure 2B), Red: Tomato, cyan: Myosin7a. Scale bars, 20 µm. C) The number of double‐positive Myo7a^+^/Tomato^+^ cells in the ear transduced by AAV‐*Atoh1*, AAV‐GPA, and AAV‐GPAS correspond to (B). *N* = 8, error bars are ±SEM. ^****^ *p* < 0.0001. D) Representative confocal images of Myosin7a and Tomato signals in the basal, middle, and apical turns of the ear injected with AAV‐*Atoh1*, AAV‐GPA, and AAV‐GPAS. Magnified areas from the GER region corresponding to (Figure 2B), Red: Tomato, cyan: Myosin7a. Scale bars, 20 µm. E) The number of double‐positive Myo7a^+^/Tomato^+^ cells in the ear transduced by AAV‐*Atoh1*, AAV‐GPA, and AAV‐GPAS, respectively, corresponds to (C). *N* = 8, error bars are ±SEM. ^**^ *p < *0.01, ^****^ *p* < 0.0001. F) Representative confocal images of Myosin7a and Otoferlin (an IHC marker) signals in the apical turns of cochleae injected with AAV‐*Atoh1*, AAV‐GPA, and AAV‐GPAS. Red: Myosin7a, cyan: Otoferlin. Scale bars, 50 µm. G) The qPCR showing the *Otof*, *Vglut3*, and *Tbx2* relative mRNA expression in the ear transduced with AAV‐*Atoh1*, AAV‐GPA, and AAV‐GPAS. Raw results from 3 replicated experiments, error bars are ±SEM. ^*^ *p < *0.05, ^**^ *p < *0.01, ^***^ *p* < 0.001.

Myosin7a^+^/Tomato^+^ HC‐like cells were detected in AAV‐GPAS injected cochleae, including the IHC region (Figure 3B), and we similarly detected the regeneration of HCs in the IHC region (**Figure** **4**A). Compared with AAV‐*Atoh1*, there were more Myosin7a^+^/Tomato^+^ HCs in the AAV‐GPA and AAV‐GPAS transduced cochleae in the IHC region (Figure 4B,C), and AAV‐GPAS induced the most Lgr5^+^ SCs to transdifferentiate into IHC‐like HCs in the apical turn of the mouse cochlea (Figure 4C). In addition, many ectopic HCs also were observed in the GER region, and some of them were labeled with tdTomato (Figure 4D). SCs in the GER region were Lgr5 negative. We therefore concluded that the new Myosin7a^+^/Tomato^+^ HCs in the GER region were derived from the differentiation and migration of Lgr5^+^ IBC/IPhCs in the IHC region (Figure 4D). We also counted the number of Myosin7a^+^/Tomato^+^ HCs in the GER region, and most HCs were lineage traced in the AAV‐GPAS transduced cochlea (Figure 4E). Otoferlin (Otof) and Vglut3 are generally considered to be the markers of IHCs in postnatal mice,^[^ ^40^, ^41^ ^]^ and previous reports in mice have shown that regenerated HCs induced by Atoh1 and GPA overexpression express Otoferlin.^[^ ^19^ ^]^ Myosin7a^+^/ Otoferlin^+^ HCs were detected in AAV‐*Atoh1*, AAV‐GPA, and AAV‐GPAS‐induced newborn HCs (Figure 4F), and the qPCR results showed that AAV‐GPAS injected cochleae expressed more Otoferlin and Vglut3 (Figure 4G). Tbx2 is a key factor that determines IHC development,^[^ ^42^ ^]^ and similarly Tbx2 was more highly expressed in both the AAV‐GPA and AAV‐GPAS groups (Figure 4G). Taken together, these data suggest that AAV‐GPA/GPAS enhanced the trans‐differentiation of SCs into OHC/IHC‐like cells in the mice.

### 2.4 AAV‐GPS Promoted the Maturation of Newborn HC‐like Cells Derived from Atoh1 Overexpression

**Figure 5:**
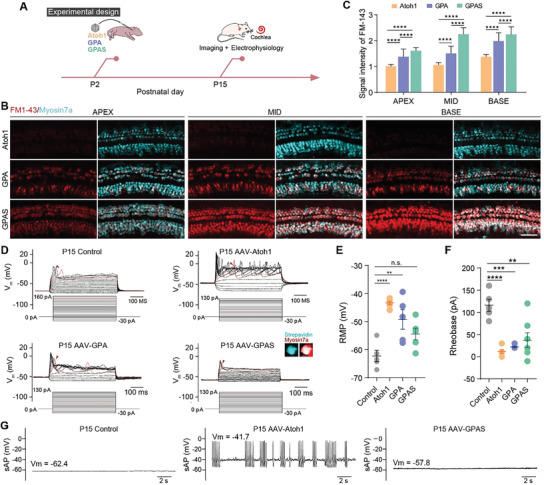
AAV‐ie‐mediated GPS overexpression enhanced the maturation of *Atoh1*‐mediated HC‐like cell regeneration in vivo. A) Experimental design diagram. All the AAVs were delivered to the mice's left ear, dose: 2.5E9 GCs per ear, then the cochleae were obtained at P15. B) The immunofluorescence images of FM1‐43 and Myosin7a signals in the basal, middle, and apical turns of the ear from P15 mice injected with AAV‐*Atoh1*, AAV‐GPA, and AAV‐GPAS. Red: FM1‐43, cyan: Myosin7a. Scale bar, 40 µm. C) The signal intensity of FM1‐43 in ear transduced by AAV‐*Atoh1*, AAV‐GPA, and AAV‐GPAS, respectively, corresponding to (B). Raw results from 3 replicated experiments, error bars are ±SEM. ^****^ *p* < 0.0001. D) Example membrane potentials of native HCs and Atoh1‐OE (overexpression), GPA‐OE, and GPAS‐OE HC‐like cells from P15 mice recorded in a current‐clamp mode. The initial calcium spike is plotted as a red line and highlighted with a red arrow. The confocal images of HC‐like cells after electrophysiological recording in the AAV‐GPAS transduced cochlea. Red: Myosin7a, HCs marker, cyan: Strepavidin, the marker of electrophysiological recording HC‐like cells. E) Dot‐plot showing the resting membrane potential (RMP) recorded in cells from each group corresponding with (D). A descending gradient in RMP was observed from the Atoh1‐OE to GPAS‐OE group that was in parallel with an ascending gradient in cell maturity. *N* = 5, error bars are ±SEM. ^**^ *p < *0.01, ^****^ *p* < 0.0001, n.s., not significant. F) Dot‐plot showing the individual and mean values of injected currents that evoked the initial spike recorded in cells from the 4 groups. *N* = 5, error bars are ±SEM. ^**^ *p < *0.01, ^****^ *p* < 0.0001. G) Example traces of spontaneous membrane potentials from native IHCs and Atoh1‐OE and GPAS‐OE HC‐like cells. A calcium‐induced burst‐spiking pattern was evident with over‐expression of Atoh1 alone, but this was abolished in both native HCs and GPAS‐OE cells.

To test the maturity and functionality of the newborn HC‐like cells induced by AAV‐GPAS/GPA/*Atoh1*, FM1‐43 staining was performed on the AAV‐transduced cochleae to characterize the MET channel function of the new HC‐like cells. The viruses were injected into the P2 wild‐type mice by RWM. Fresh cochleae were transiently infiltrated in FM1‐43 dye for 45 s after dissection at P15 (**Figure** **5**A). As predicted, uptake of FM1‐43 dye in new HCs was detected in all AAV‐GPAS/GPA/*Atoh1* transduced cochleae. The strongest FM1‐43 signals were observed in regenerated HC‐like cells from the AAV‐GPAS transduced cochlea, followed by the AAV‐GPA groups (Figure 5B,C), indicating that AAV‐GP strengthened the expression and permeability of the MET channels in AAV‐*Atoh1* induced new HC‐like cells. In addition, we detected the morphology of the regenerated HC‐like cells by scanning electron microscope (SEM), the images showed the stereocilia of the regenerated HC‐like cells were arranged in the “V” shape similar to the normal stereocilia (Figure S11, Supporting Information).

Next, we performed electrophysiological recordings of native HCs and HC‐like cells by whole‐cell patch to evaluate whether GP/GPS‐overexpressing HC‐like cells were more similar to native HCs. We found that, unlike the multiple calcium‐spikes observed in *Atoh1* alone or triple GPA‐induced HC‐like cells that mirrored neonatal IHCs, HC‐like cells induced by GPAS behaved much like adult P15 IHCs with characteristics of evoked calcium spikes requiring large currents and saturated spiking in the form of singlets (Figure 5D–F). Additionally, we patched these induced HC‐like cells and native HCs in gap‐free mode to record their spontaneous firings in a current clamp and found that AAV‐GPAS‐induced IHCs exhibited weak and small spontaneous fluctuations that were quite similar to those of native HCs. However, the Atoh1‐induced IHC‐like cells spontaneously generated burst‐firings that were in line with the neonatal IHC signature (Figure 5G). Taken together, these results showed that the combined regulation of GPA and GPAS in cochlear progenitors can promote the further maturation of newborn HC‐like cells and indicate the application prospect of AAV‐mediated multigene synergistic regulation in the regeneration of functional HCs for the clinical treatment of hearing loss.

### 2.5 AAV‐GPAS Promoted the Regeneration of HC‐like Cells in P7 Mice

**Figure 6:**
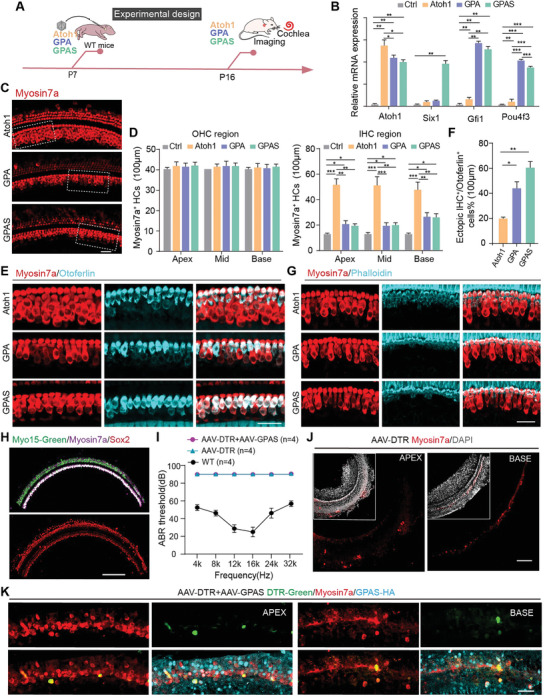
AAV‐ie‐mediated Atoh1, GPA, and GPAS effectively regenerated HC‐like cells in P7 mice. A) Experimental design diagram. All the AAVs were delivered to the mice's left ear, dose:2.5E9 GCs per ear. B) The qPCR showing the relative mRNA expression of *Atoh1*, *Gfi1*, *Pou4f3*, and *Six1* in the cochleae injected with AAV‐control, AAV‐*Atoh1*, AAV‐*Six1*, AAV‐GPA, and AAV‐GPAS. Raw results from 3 replicated experiments. Error bars are ±SEM. ^*^ *p < *0.05, ^**^ *p < *0.01, ^***^ *p* < 0.001. C) The immunofluorescence images of Myosin7a signals in the basal and middle turns of the ear injected with AAV‐*Atoh1*, AAV‐GPA, and AAV‐GPAS, respectively at P16 mice. Red: Myosin7a. Scale bar, 30 µm. D) The number of Myosin7a in the AAV‐*Atoh1*, AAV‐GPA, and AAV‐GPAS injected ear in (C). *N* = 5, error bars are ±SEM. ^*^ *p < *0.05, ^**^ *p < *0.01, ^***^ *p* < 0.001. E) The immunofluorescence images of Myosin7a and Otoferlin signals in the basal and middle turns of the ear injected with AAV‐*Atoh1*, AAV‐GPA, and AAV‐GPAS, at P16 mice, respectively. Magnified areas corresponding to (C). Red: Myosin7a, cyan: Otoferlin. Scale bar, 30 µm. F) The percentage of the ectopic double‐positive IHC^+^/Otoferlin^+^ cells in the cochleae corresponding to (E). *N* = 5, error bars are ±SEM. ^*^ *p < *0.05, ^**^ *p < *0.01. G) Representative confocal images of Myosin7a and Phalloidin signals in the apical to middle turns of the ear injected with AAV‐*Atoh1*, AAV‐GPA, and AAV‐GPAS, respectively, at P16 mice. Red: Myosin7a, cyan: Phalloidin. Scale bar, 30 µm. H) Representative confocal images of Myosin7a, Sox2, and Myo15‐Green signals in the apical turn of ear injected with Anc80L65‐Myo15‐mNeonGreen at P2 WT mice. Red: Sox2, Magenta: Myosin7a. Scale bar, 200 µm. I) The ABR results of WT mice, AAV‐DTR (AAV‐Myo15‐DTR‐mNeonGreen), and AAV‐DTR+AAV‐GPAS injected ear at P30 mice. N = 4, error bars are ±SEM. J) Representative confocal images of Myosin7a and DAPI signals in the apical turn of the ear injected with AAV‐DTR at P30 mice. Red: Myosin7a, grey: DAPI. Scale bar, 200 µm. K) Representative confocal images of Myosin7a, HA, and DTR‐Green signals in the apical and basal turns of ear injected with AAV‐DTR+AAV‐GPAS at P30 mice. Red: Myosin7a, cyan: HA, Green: AAV‐DTR‐mNeoNGreen. Scale bar, 40 µm.

A previous report showed GPA overexpression could induce the regeneration of HCs, while Atoh1 overexpression alone could not regenerate HCs in P8 tamoxifen injected and P15 harvested Sox9‐CreER/Rosa26^GPA^ mice.^[^ ^19^ ^]^ This study used transgenic mouse models to explore the role of Atoh1 or GPA in HC regeneration. Here, we used AAV delivery to study the effect of GPAS on HC regeneration in the elder mice. We used AAV to deliver *Atoh1*, GPA, and GPAS into the cochleae of P7 mice and harvested them at P16 (**Figure** **6**A). The expression of these 4 exogenous genes at the transcriptome level was measured after AAV injection by the qPCR experiment, the results showed the expression of all 4 genes increased dramatically (Figure 6B), indicating the successful expression of AAV‐ie‐mediated gene transfer into the P7 mice cochlea. The immunofluorescence staining results indicated that AAV‐*Atoh1*/GPA/ GPAS could induce HC‐like cell regeneration in the cochleae at P16 (Figure 6C,D). Most of these regenerated HC‐like cells were located in the IHC region. So next, we detected the expression of Otoferlin, and found that some of the regenerated HC‐like cells in the 3 groups expressed Otoferlin (Figure 6E). The quantitative analysis that the greatest percentage of ectopic IHC^+^/Otoferlin^+^ double‐positive HCs was seen in the AAV‐GPAS‐induced ectopic HCs (Figure 6F). Furthermore, we examined the stereocilia of regenerated HC‐like cells by Phalloidin staining, and the stereocilia in the AAV‐ *Atoh1*/GPA/ GPAS induced new HC‐like cells had weaker Phalloidin signals compared to the native HCs (Figure 6G). To corroborate the findings of the preceding article,^[^ ^19^ ^]^ Sox9^creER^/tdTomato mice were employed for lineage tracing to validate the source of regenerated HC‐like cells subsequent to AAV injection at P7. The outcomes demonstrated a predominant derivation of regenerated HC‐like cells from Sox9^+^ SCs in AAV‐Atoh1/GPA/GPAS transduced cochlea (Figure S12A,B, Supporting Information).

Next, we conducted to elucidate the functionality of these regenerated HC‐like cells in a DTR injury model. Previous findings demonstrated specific expression of EGFP in HCs driven by the HC‐specific promoter Myo15,^[^ ^43^ ^]^ our results revealed that AAV‐Myo15‐mNeonGreen efficiently infected HCs in neonatal mice with the 100% infection rate, and SCs remained unaffected (Figure 6H). We injected AAV‐Myo15‐DTR‐mNeonGreen into P1 WT mice and administered diphtheria toxin at P4, resulting in nearly complete loss of hearing and HCs at P30 (Figure 6I,J). Then, we administered AAV‐GPAS at P7, and the auditory brainstem response (ABR) data indicated that AAV‐GPAS did not restore hearing function impaired by DTR‐induced injury in P30 mice (Figure 6I). However, immunofluorescence results demonstrated that AAV‐GPAS induced HC‐like cell regeneration after DTR injury (Figure 6K). Taken together, these results showed that AAV‐*Atoh1*/GPA/GPAS could induce HC‐like cell regeneration in the cochleae of P7 mice, and AAV‐GPAS promotes the formation of new HC‐like cells after DTR‐induced injury.

## 3 Discussion

In mammals, cochlear HCs lack the ability to regenerate spontaneously, and loss of HCs will lead to permanent hearing loss. Cochlear HCs are divided into 2 different types, with OHCs being responsible for amplifying sound signals^[^ ^38^, ^44^ ^]^ and IHCs being responsible for transmitting sound signals.^[^ ^45^, ^46^ ^]^ Functional HC regeneration is considered to be an effective therapy for hearing loss, and in our experiments, we used AAV to drive the expression of exogenous GPAS in cochlear precursor cells. AAV‐ie is a safe AAV with high infection efficiency (>80%) for SCs, including HeCs, DCs, OPCs, IPCs, IPhCs, and IBCs, that was developed by our partners in 2019,^[^ ^24^ ^]^ and it is currently the most efficient AAV for infection of these SCs. Therefore, AAV‐ie‐mediated gene regulation has obvious advantages in the trans‐differentiation of SCs into HCs. Compared with using transgenic mice, AAV‐ie is straightforward and convenient, and our results represent an important step toward integrating AAV‐mediated gene regulation and new HC regeneration into auditory organ reconstruction in vivo. Currently, studies have reported the successful restoration of hearing function in DFNB9 patients through AAV‐mediated gene therapy, highlighting its significant potential for the treatment of deafness.^[^ ^47^, ^48^ ^]^ AAV‐Atoh1/GPA/GPAS could regenerate massive iHCs in the GER and IHC region, while fewer HCs were regenerated in the OHC region. Previous studies have shown that SCs induced by Atoh1 activation that could directly transdifferentiate into regenerating HCs were mainly IPCs, DCs, and IPhCs/IBCs,^[^ ^9^, ^10^, ^16^ ^]^ whereas in the lateral non‐sensory epithelial region, SCs capable of regenerating HCs were predominantly DCs, which may contribute to the low number of regenerating HCs in OHC region.

Currently, a variety of regeneration strategies are applied to generate new functional HCs. *Atoh1* is a classic HC differentiation gene^[^ ^7^ ^]^ that can direct the differentiation of cochlear sensory epithelial precursor cells into HCs through cellular programming.^[^ ^49^, ^50^, ^51^, ^52^ ^]^ The overexpression of Atoh1 results in the formation of HCs and SCs in the non‐sensory region of the cochlea^[^ ^53^ ^]^ or the formation of ectopic HCs,^[^ ^54^, ^55^, ^56^, ^57^ ^]^ and Atoh1 combined with other signals such as Wnt,^[^ ^37^, ^58^ ^]^ Notch,^[^ ^59^ ^]^ etc., enhance Atoh1‐mediated HC regeneration potential. However, these newly generated HCs were in the early stage of HC development and did not have the same functionality as mature HCs. *Atoh1* is the upstream control gene of *Pou4f3* and *Gfi1*, and the activation of *Atoh1* in cochlear progenitors initiates the expression of Pou4f3^[^ ^60^ ^]^ and subsequent activation of Gfi1.^[^ ^14^ ^]^ Therefore, Pou4f3 and Gfi1 are considered the main factors that promote HC maturation. The latest research showed that Gfi1 and Pou4f3 can promote the regeneration function of Atoh1 alone and enhance the relative maturation of the physiological functions of new HCs.^[^ ^17^, ^18^ ^]^ We reached similar conclusions using AAV‐ie‐mediated gene co‐regulation. Compared with AAV‐*Atoh1* alone, the induced HCs in the AAV‐GPA‐injected cochlea showed a relatively more mature state. In addition, we found that the addition of *Six1* could further guide the maturation process of new HCs. *Atoh1* is a downstream target gene of *Six1*, and co‐regulation of *Six1* enhances GPA‐induced HC regeneration and the maturation of regenerated IHCs. Nevertheless, *Six1* is not able to enhance Atoh1‐induced HC regeneration. In addition, researchers found that Atoh1 could induce the differentiation of cultured mouse embryonic fibroblasts (MEFs) into HCs, while Pou4f3, Gfi1 and Six1 expression alone failed to produce HCs from MEFs.^[^ ^61^ ^]^ At the same time, they screened 14 other transcription factors and found that GPAS had the greatest effect on MEF to HC conversion.^[^ ^61^ ^]^ Together, these findings suggest that the regulation of HC regeneration is the result of multi‐gene synergism, among which GPAS is a powerful new combination for inducing functional HC regeneration.

Cochlear SCs are considered to be HCs precursors, and AAV‐GPAS mediates the regeneration of HCs mainly through SC trans‐differentiation. Sox2 as the Sox family transcription factor is expressed in inner ear precursor cells, and as different cochlear epithelial cell types have specific differentiation models in the otic placode, Sox2 shows a distinct expression pattern, maintained in adult SCs but down‐regulated in HCs. The activity of Six1 is critical for the down‐regulation of Sox2 in differentiated HCs during cochlear development. The loss of *Six1* alters the spatial and temporal patterns of Sox2 expression in the differentiated cochlea, and high expression of Sox2 was seen in all cells of the cochlea with the *Six1* conditional knockout.^[^ ^62^ ^]^ Because Atoh1‐induced HCs were derived from SC trans‐differentiation, we measured the expression level of Sox2 in regenerated new HCs. Although Aoh1 can induce mass SCs to transdifferentiate into HCs, the expression of Sox2 in newly formed HCs is still at a very high level (Figure S4, Supporting Information), which is one of the specific indications that Atoh1 alone cannot induce mature HCs. After the addition of GP, the expression of Sox2 was significantly decreased, which was further enhanced by the addition of Six1, which reduced Sox2 expression in regenerated HCs. The expression of Sox2 could barely be detected in some GPAS‐induced regenerated HCs (Figure S4, Supporting Information), indicating that the addition of GPS synergistically promoted the reduction of Sox2 expression in Atoh1‐induced newborn HCs, which greatly improved HC maturation. These results were consistent with those obtained from MEFs in vitro,^[^ ^21^ ^]^ and together these results suggest that although *Six1* is an upstream regulator of genes critical for HC differentiation and development, it does not by itself trigger HC‐oriented fate differentiation.

Previous studies showed that Supervillin is an F‐actin cross‐linked protein that is a major component of the cuticular plate of HCs in the mouse cochlea and vestibule.^[^ ^27^ ^]^ And the previous report showed that *Myo6*, an HC marker, is a target gene of Zeb1,^[^ ^28^ ^]^ and Zeb1 might therefore play an important role in HC regeneration.^[^ ^29^ ^]^ Chd7 is localized to auditory neurons and HCs, and embryonic loss of Chd7 leads to sensorineural hearing loss.^[^ ^30^ ^]^ Ankrd6 is asymmetrically located in the sensory organs of the inner ear, which is characteristic of conserved core planar cell polarity complex components, and the absence of mAnkrd6 results in planar cell polarity defects in the sensory organs of the inner ear.^[^ ^31^ ^]^ In our sequencing and qPCR data, these genes were highest in the AAV‐GPAS group, thus changes in these genes may be responsible for the maturation of AAV‐GPAS‐induced HC‐like cells.

It has been reported that Six1 knockdown alters the proliferation ability of cochlear progenitors during HC development in embryonic day 14.5 mice.^[^ ^62^ ^]^ We injected the virus at P1 followed by EdU at P2‐4 to label the dividing inner ear stem cells. However, we did not observe any EdU^+^/Myo7a^+^ cells in the AAV‐GPAS‐infected cochlear epithelium (Figure S10, Supporting Information), indicating that Six1 overexpression did not induce SCs to trans‐differentiate to the HCs in young mice and that elder inner ear progenitors in young mice are not as strongly regulated by Six1 as they are in the embryo. Activation to produce more inner ear progenitors may require the combined action of more transcription factors that regulate the cell cycle.

The electrophysiological results revealed a significantly lower level of maturation in HC‐like cells regenerated through P7 injection of AAV‐GPAS compared to those regenerated through P2 injection of AAV‐GPAS (Figure S12C, Supporting Information). Previous studies have demonstrated that cochlear developmental genes, such as Atoh1, exhibit greater accessibility in HCs and SCs during P1 compared to P8 mice, which display more pronounced methylation in SCs and inhibit HC regeneration in P8 mice.^[^ ^63^ ^]^ This may account for the reduced maturation of regenerated HCs following AAV‐GPAS injection after P7, thereby impeding the restoration of auditory function after DTR injury. Despite the existence of various strategies to promote HC regeneration by multiple genes,^[^ ^19^, ^20^, ^22^ ^]^ there remains a dearth of research on restoring hearing function through regenerative HCs in cases of HC damage. This suggests that achieving hearing recovery after injury is a complex process that demands further investigation.

A previous report showed that Atoh1 overexpression alone could not regenerate HCs in Sox9‐CreER/ Rosa26^GPA^ mice injected with tamoxifen at P8 and harvested at P15.^[^ ^19^ ^]^ However, our data showed that AAV‐*Atoh1* could regenerate new HCs in the cochlea in mice injected at P7 and harvested at P16. This inconsistency with previous studies may be due to the following 2 reasons. First, Atoh1 may have a dose‐dependent effect. In our data, Atoh1 expression mediated by AAV might be higher than that of transgenic Sox9‐CreER/ Rosa26^GPA^ mice. Second, overexpression of AAV‐Atoh1 leads to in situ HCs injury, which might promote Atoh1‐induced SC to HC trans‐differentiation, because previous studies have shown that HC injury can promote SC to HC trans‐differentiation.^[^ ^5^ ^]^ Similarly, our results showed Atoh1 overexpression in adult mice could damage HCs, we injected AAV‐*Atoh1*, AAV‐GPA, and AAV‐GPAS into adult mice cochlea, instead of HC regeneration, HC loss occurred (Figure S13, Supporting Information). In addition, AAV‐GPAS overexpression could not recover hearing and regenerate HCs in the neomycin‐damaged HCs model (Figure S14, Supporting Information). The limited infective efficiency of AAV‐ie on adult SCs^[^ ^24^ ^]^ may underlie the failure of AAV‐GPAS to induce HC regeneration in adult mice and restore hearing function in adult mice with neomycin‐induced damage. And, there is no AAV vector that can efficiently infect adult mouse SCs, therefore, the identification and screening of suitable AAV vectors will be pivotal for future gene therapy studies in adult mice.

Here we used the transcription factor cocktails to induce the regeneration of relatively more mature HCs. The synergistic regulation of multiple factors significantly improved the HC regeneration efficiency and the ability for directed differentiation of IHCs and OHCs compared with Atoh1‐induced HCs. This regulation was achieved through AAV‐mediated overexpression of exogenous genes.

## 4 Experimental Section

### Animals and Genotyping

In this paper, the different mice including wild‐type C57BL/6J mice, transgene Lgr5‐EGFP^creERT2^ mice (Jackson Laboratory), Sox9 ^creERT2^ mice (Jackson Laboratory), and Rosa26‐CAG‐tdTomato mice (Jackson Laboratory) were used. In P1‐2 mice, the Cre recombination enzyme was activated by intraperitoneal injection of tamoxifen (Sigma, T5648), and the dose was controlled at 0.3 mg/4 g. Diptheria toxin (List Biologicals Lab) was intraperitoneally injected into P4 mice at a dose of 10 ng g^−1^. The genomic DNA of the Lgr5^CreER^/Tomato^loxp/+^, or Sox9^CreER^/Tomato^loxp/+^ was harvested by alkali lysis with NaOH, and the DNA was used as the PCR template with the primers and 2× Taq PCR Master mix buffer (Protein Biotechnology, PC106) and amplified by a PCR instrument (Bio‐Rad, T100). The genotyping primers and sequences used for mice are listed in Table S1 (Supporting Information). The PCR products were isolated by DNA gel electrophoresis. In this paper, all mice experiments were completed according to the standard permitted by the Use Committee of Southeast University and Animal Care and used a minimum number of animals (No.20210606001).

### AAV Viruses Packaging

The corresponding transgenes Myo15‐mNeonGreen, DTR‐mNeonGreen, *Six1*‐HA, *Atoh1*‐HA, *Gfi1*‐P2A‐*Pou4f3*‐HA, and *Atoh1*‐P2A‐*Six1*‐HA) were inserted into the AAV skeleton plasmid. In this study, except Anc80L65 for Myo15‐mNeonGreen and DTR‐mNeonGreen, the capsid of the remaining AAV viruses used AAV‐ie as the capsid vector, then the target plasmids, Anc80L65/AAV‐ie capsid plasmid, and helper plasmid were co‐transfected into HEK‐293T cells. The cells and medium were collected after 96 h, and chloroform was used to lyse the harvested cells. AAVs were purified as previously described.^[^ ^64^, ^65^ ^]^ The titer of AAVs was examined by the qPCR using the SYBR system (Vazyme, Q712‐02), and the qPCR primer sequences are shown in Table S1 (Supporting Information).

### AAV Injection via the RWM

The AAV injection protocol used in this study is according to the previous report.^[^ ^24^ ^]^ The P1‐2, or P7 newborn mice were anesthetized at low temperatures, and the RWM was observed under an optical microscope. For every mouse, the AAV volume was controlled at 1.5 µL, and the AAVs were injected into the cochlea via the RWM. The wounds of the AAV‐injected mice were closed with the tissue adhesive (3 m Vetbond, 1469SB), and the mice were moved to a heating pad at 37.5°C until their body temperature fully recovered, after which the mice were returned to their mother for follow‐up feeding.

### AAV Injection via the Posterior Semicircular Canal (PSCC) in Adult Mice

Following anesthesia, the hair surrounding the left ear of mice was meticulously cleaned. Then a small incision was cut behind the left ear, and the fat and tissue were stripped away to expose the PSCC, next a small hole was punched in the PSCC, the virus extracted from the glass electrode in advance was injected through the hole, and the wound was stitched. For every mouse, the AAV volume was controlled at 2 µL, and the mice were moved to a heating pad at 37.5 °C until their body temperature fully recovered.

### Immunofluorescence Staining

The cochlear sample was dissected in cold HBSS, and fixed with 4% paraformaldehyde (PFA, Sangon Biotech, A500684‐0500) for 2–3 h at room temperature (RT). The cochleae from mice older than P7 needed to be immersed in EDTA buffer (0.5 M Biosharp, BL518A) for 2–8 h at RT. The basilar membrane was collected under an optical microscope. Then transferred to slides pre‐coated with Cell‐Tak (Corning, 354 240). The samples were blocked for 1–2 h at RT with a blocking medium, and the primary antibody was supplied to the cochlear samples overnight at 4 °C. And the primary antibodies have Myosin7a (1:1000 dilution, Proteus Bioscience, 256 790), HA (1:200 dilution, Roche, 11 867 423 001), Sox2 (1:200 dilution, R&D systems, 245 610), Otoferlin (1:200 dilution, Abcam, ab53233), Slc26a5 (1:200 dilution, Santa Cruz, sc‐22692). After incubation with the corresponding secondary antibodies, cochlear samples were coated with DAKO anti‐fluorescence quenching medium (DAKO, S3023). The laser confocal microscope (Zeiss LSM 700 & 900) was used to capture the images.

### 10× Chromium Single‐Nucleus RNA Sequencing: Nucleus Isolation and Quality Control

The complete single‐cell RNA sequencing was performed by the 10× Genomics method, essentially as described previously.^[^ ^66^ ^]^ Briefly, cell suspensions were loaded in every channel with a 5000‐cell target output for each sample, and sample processing was performed according to the manufacturer's protocol. Cell Ranger (3.1.0) with default parameters was used to compare and quantify the dataset. The data was compared to the mouse reference genome (mm10) by STAR aligner to generate the gene expression matrix, and the cell ranger count function was used to quantify the sample‐specific FASTQ file. Then the filtered gene expression matrix was used for down‐stream analysis.

### snRNA‐seq Data Analysis and Cell‐Type Confirmation

Down‐stream analysis for single‐nucleus RNA‐seq data was processed by the Seurat (version 4.1.1) single‐cell toolkit in R (version 4.1.0). Cells containing more than 500 genes and fewer than 15000 genes were included for subsequent analysis. The data with the *SCTransform* workflow and canonical correlation analysis for the integration of the AAV‐*Atoh1*, AAV‐GPA, and AAV‐GPAS groups was normalized and scaled. Marker genes for each cluster were confirmed with the ROC test (min.pct = 0.1, AUROC threshold = 0.7) using the *Find‐All‐Markers* function. The 4 HC marker genes *Slc26a5*, *Slc17a8*, *Tmc1*, and *Atoh1* were used as references for the dynamic phases of OHCs and IHCs. For example, relatively higher expression of Slc26a5 and Tmc1 indicate mature OHCs, higher expression of Slc17a8 and Tmc1 indicate mature IHCs, and exclusively higher expression of Atoh1 indicates mostly immature HCs. Venn graphs were produced using the Euler package v6.0.0 to show the relationships of differentially expressed genes across 3 groups for a given subtype (various phases of HC state). The heatmaps of differentially expressed genes for all subtypes under a given group were produced by down sampling every subtype to 50 random nuclei in each group.

### Pseudotime Analysis

HC trajectories were generated in the Monocle v2.0 R package (https://github.com/cole‐trapnell‐lab/monocle‐release). Metadata was imported into Monocle with the “importCDS” function, and pseudotime‐based trajectories were used for ordering cells across the 3 groups using the “orderCells” function. The Monocle's differential expression analysis confirmed genes that varied significantly across groups along the pseudotime‐axis by using a p‐value. The top 100 differentially expressed genes were used to generate a heatmap through pseudotime‐based differential expression analysis.

### Whole‐Cell Patch‐Clamp Recording

All experiments were completed on the apical region of HCs or AAV‐infected HC‐like cells in P15‐16 mice of both sexes. The regenerating HCs that were examined in the electrophysiology experiments were located in rows 1–3 below the in situ HCs. Cochleae were obtained in dissection solution (10 mm D‐glucose (325 mOsm, pH 7.25), 141.7 mm NaCl, 5.36 mm KCl, 0.5 mm MgSO4, 10 mm HEPES, 0.1 mm CaCl2, 1 mm MgCl2, and 3.4 mm L‐Glutamine). When recording, cochlea was maintained in an external 1.3 Ca solution (5.6 mM D‐glucose (325 mOsm, pH 7.35), 0.9 mm MgCl2, 5.8 mm KCl, 10 mm HEPES, 0.7 mm NaH2PO4, 1.3 mm CaCl2, and144 mm NaCl).

HCs were observed with an inverted microscope with a 60×1.00NA water‐immersion objective and differential interference contrast. Whole‐cell patch clamp recordings were carried out using an Olympus microscope (BX51WI) and AXON 7500B amplifiers. Patch pipettes (10–15 MΩ) contained 0.1 mm CaCl2, 135 mm KCl, 5 mm HEPES‐KOH (325 mOsm, pH 7.35), 2.5 mm Na2ATP, 5 mm EGTA‐KOH, and 3.5 mM MgCl2. During recordings, HCs and HC‐like cells were maintained at a holding potential of −70 mV. Recordings were low‐pass filtered at 2 kHz, and data analysis was performed using Clamfit10 and Graphpad7.

### Western Blotting

Cochlear samples were harvested from P15 mice injected with AAV in the left ear at P1‐2. The cochlear samples were lysed with RIPA lysis buffer (Sangon Biotech, C500008) and protease inhibitor cocktail (Roche, 0 469 313 2001) and then homogenized. The protein supernatant was obtained by high‐speed centrifugation. The supernatant was diluted with 5× SDS loading buffer and then boiled at 100 °C for 8–10 min. The protein was separated by 10% Bis‐tris PAGE (GenScript, M01115C) in the running buffer, and the protein bands were transferred onto PVDF membranes (Millipore, ISEQ00010) in the transfer buffer. The skim milk was used to block PVDF membranes for 1–2 h at RT, and primary antibodies were added at 4 °C overnight on a shaker. The primary antibodies were Atoh1 (1:1000 dilution, Santa Cruz, sc‐136173), Pou4f3 (1:1000 dilution, Santa Cruz, sc‐81980), Gfi1 (1:1000 dilution, Santa Cruz, sc‐373960), Six1 (1:1000 dilution, Sigma, HPA001893), and GAPDH (1:2000 dilution, Abcam, ab8245). GAPDH acted as the internal control gene in all of the experiments. On the next day, the secondary antibodies were incubated at RT for 1 h, and images were acquired with Chemi Capture (Tanon, 5200).

### Organoid Culture

All experimental operations were performed according to the previous protocol.^[^ ^64^ ^]^ In this experiment, P1‐2 wildtype mice were chosen to obtain the basilar membrane, and the single‐cell suspension was obtained by enzymatic lysis (0.25% Trypsin‐EDTA, Thermo Fisher Scientific, 25200‐056). The single‐cell suspension was hybridized with Matrigel (Corning, 356 234) and added to the 24‐well plate at 6000 cells per well. The single cells were cultured in the expansion medium (DMEM/F12, 2% B‐27 (Thermo Fisher), 1% N2 (Thermo Fisher), VPA (1 mM, Sigma), IGF (50 ng mL^−1^, Sigma), 616 452 (2 µM, Sigma), EGF (20 ng mL^−1^, Sigma,), b‐FGF (10 ng mL^−1^, Sigma), CHIR99021 (3 µM, Sigma), and 1% ampicillin (Sigma)) for 10 days. Then cultured in a differentiation medium (DMEM/F12, 2% B‐27, 1% N2, LY411575 (5 µM, Sigma), CHIR99021, and 1% ampicillin) for 10 days. AAVs were supplied 2 days ago of culture at a dose of 2e10 GCs per well.

### ABR Experiment

All experimental mice were anesthetized with sodium pentobarbital (100 ug g^−1^). The tone‐burst ABR (4, 8, 12, 16, 24, and 32 kHz) and click ABR were examined to analyze the mice hearing. Next, the positive electrode was inserted subcutaneously in the middle suture of the mouse head, the negative electrode was inserted subcutaneously behind the test ear, and the reference electrode was enrolled subcutaneously behind the contralateral ear. The closed‐field test was used for the injected ear and contralateral ear by the TDT workstation (RZ6, Tucker‐Davis Technologies).

### RNA Extraction and qPCR Analysis

The cochlear and organoid samples were collected in cold HBSS followed by treatment with Trizol (Thermo Fisher Scientific, 15596‐018), and the RNA was extracted with chloroform and isopropanol. The reversed kit (Thermo Fisher Scientific, 0 113 4583) was used to reverse RNA to cDNA on a PCR instrument. The cDNA was used as the template, and qPCR primers and SYBR (Roche, 4 913 850 001) were mixed for the qPCR‐detected experiment with *Gapdh* acting as the internal control gene in the experiment. The qPCR primer sequences are listed in Table S1 (Supporting Information).

### Scanning Electron Microscopy

The P1‐2 wildtype mice were injected in the left ear with AAVs, and the cochleae were obtained from P15 mice in the cold HBSS and then fixed with 2.5% at 4 °C for 12  h. The cochleae were decalcified in EDTA for 5–8 h at 4 °C. And, then divided into the basal, middle, and apical turns under an optical microscope. The basilar membrane was incubated with 2% osmium tetroxide at 4 °C for 2–4 h, and after washing with ddH_2_O the samples were dehydrated in an ethanol gradient from 30% to 100%. After critical point drying (Leica, EMCPD300) and ion sputtering gilding, the cochlear samples were imaged on a scanning electron microscope (JSM‐7800F prime).

### Statistical Analysis

In this study, all raw confocal images were exported in. tif format using the microscope LSM 700 & 900 (Zeiss, Inc.). Then the raw images were revised with the Fiji software (Fiji, Inc.), and statistical analyses were completed by using the GraphPad Prism 7.0 software. For statistical analysis, the double positive Myosin7a^+^/Tomato^+^ cells were counted per 100 µm in the images with the Fiji software. For the analysis of the fluorescence intensity of Sox2, the cell boundary was marked, the fluorescence intensity was measured by the Fiji software, and the statistical results were displayed using the GraphPad software. The Student's *t*‐tests or one‐way ANOVA were performed to measure the statistical difference from at least 3 independent experiments, and results were presented as mean ± SEM. The value of *p* < 0.05 was considered statistically significant.

## Conflict of Interest

J.Q. and F.T. have filed a patent on the use of AAV‐ie for gene therapy in the inner ear. The authors declare no other conflict of interest.

## Author Contributions

L.Z., X.C., X.W., Y.Z., and Y.F. contributed equally to this work. L.X., F.T., J.Q., and R.C. conceived and designed the experiments. L.Z., X.C., X.W., Y.Z., and Y.F. performed most of the experiments. L.Z. injected all AAVs into the cochleae. L.Z., Y.Z., and Y.F. performed the immunofluorescence staining and image capture. X.C. and X.W. obtained the electrophysiological data and analyzed the single‐cell nuclear sequencing data. L.Z. and X.C. contributed to the data analysis. Y.Z. completed the AAV packing and ABR measurement. X.G., Z.Z., N.L., and Q.S. helped with the experiments and the data analysis. X.G. helped complete the revision work. L.X. oversaw the completion of the revision process and refined the entire manuscript. J.Q., L.Z., and X.C. discussed the data analysis, interpretation, and presentation and wrote the manuscript with contributions from all authors.

## Supporting information

Supporting Information

## Data Availability

The data that support the findings of this study are available in the supplementary material of this article.
