# Correction to “AAV‐Mediated Gene Cocktails Enhance Supporting Cell Reprogramming and Hair Cell Regeneration”

**DOI:** 10.1002/advs.202416025

**Published:** 2025-01-10

**Authors:** Liyan Zhang, Xin Chen, Xinlin Wang, Yinyi Zhou, Yuan Fang, Xingliang Gu, Ziyu Zhang, Qiuhan Sun, Nianci Li, Lei Xu, Fangzhi Tan, Renjie Chai, Jieyu Qi

*Adv. Sci*. **2024**, *11*, e2304551.

http://doi.org/10.1002/advs.202304551

Description of the errors:

In the original published paper, we found that the “Apex” image of the AAV‐Atoh1 group in Figure 5A was improperly used. The corrected figures are shown below.

Corrected Figure 5A:


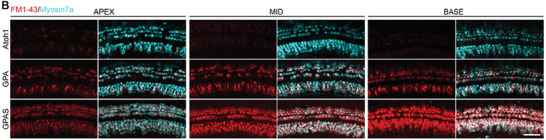


This correction does not affect the overall findings and conclusions of this paper. We apologize for this error.

