# Supplementary material for: AAV‐mediated Gene Cocktails Enhance Supporting Cell Reprogramming and Hair Cell Regeneration
Source: Adv Sci (Weinh). 2024 May 29;11(29):2304551. doi: 10.1002/advs.202304551 (PMC11304307; doi:10.1002/advs.202304551)
Supplement: Supplementary file 1 — Supporting Information [file ADVS-11-2304551-s001.docx]

Supplementary Materials for

**AAV-mediated Gene Cocktails Enhance Supporting Cell Reprogramming and Hair Cell Regeneration**

Liyan Zhang^1#^, Xin Chen^1#^, Xinlin Wang^1#^, Yinyi Zhou^1#^, Yuan Fang^1#^, Xingliang Gu^1^, Ziyu Zhang^1^, Qiuhan Sun^1^, Nianci Li^1^, Lei Xu^2,3*^, Fangzhi Tan^1*^, Renjie Chai^1,4,5,6,7*^, Jieyu Qi^1,4,5*^.

^*^Corresponding author: sdphxl@126.com, tanfangzhi@163.com, [renjiec@seu.edu.cn](mailto:renjiec@seu.edu.cn), and [jieyuqi@seu.edu.cn](mailto:jieyuqi@seu.edu.cn).

The PDF file includes:

Fig. S1. AAV-ie-NLS-mNeonGreen was highly effective at infecting HCs and SCs in mice.

Fig. S2. AAV-ie-mediated two AAVs can efficiently infect the same cells.

Fig. S3. Forced Six1 expression in the cochlea was not capable of regenerating HCs.

Fig. S4. Sox2 staining in AAV-*Atoh1*, AAV-GPA, and AAV-GPAS-transduced mice.

Fig. S5. Single-nucleus RNA sequencing of cochlear epithelium with over-expression of *Atoh1*.

Fig. S6. Single-nucleus RNA sequencing of cochlear epithelium with co-expression of *Gfi1*, *Pou4f3*, and *Atoh1*.

Fig. S7. Single-nucleus RNA sequencing of cochlear epithelium with co-expression of *Gfi1*, *Pou4f3*, *Atoh1*, and *Six1*.

Fig. S8. Single-nucleus RNA sequencing of AAV-infected HCs with co-expression of one, three, or four transcription factors.

Fig. S9. Go analysis of differential genes and transcription factors in the AAV-*Atoh1*, AAV-GPA, and AAV-GPAS group.

Fig. S10. EdU staining in AAV-GPA and AAV-GPAS-transduced cochleae.

Fig. S11. Scanning electron microscope images of regenerated HCs.

Fig. S12. Regenerated HC source and maturity of P7 mice after AAV-GPAS injection.

Fig. S13. AAV-ie-mediated Atoh1, GPA, and GPAS incapably regenerated HCs in P30 mice.

Fig. S14. AAV-GPAS injection failed to restore hearing and regenerate HCs in neomycin damaged model.

Table S1. Primers used in this manuscript.
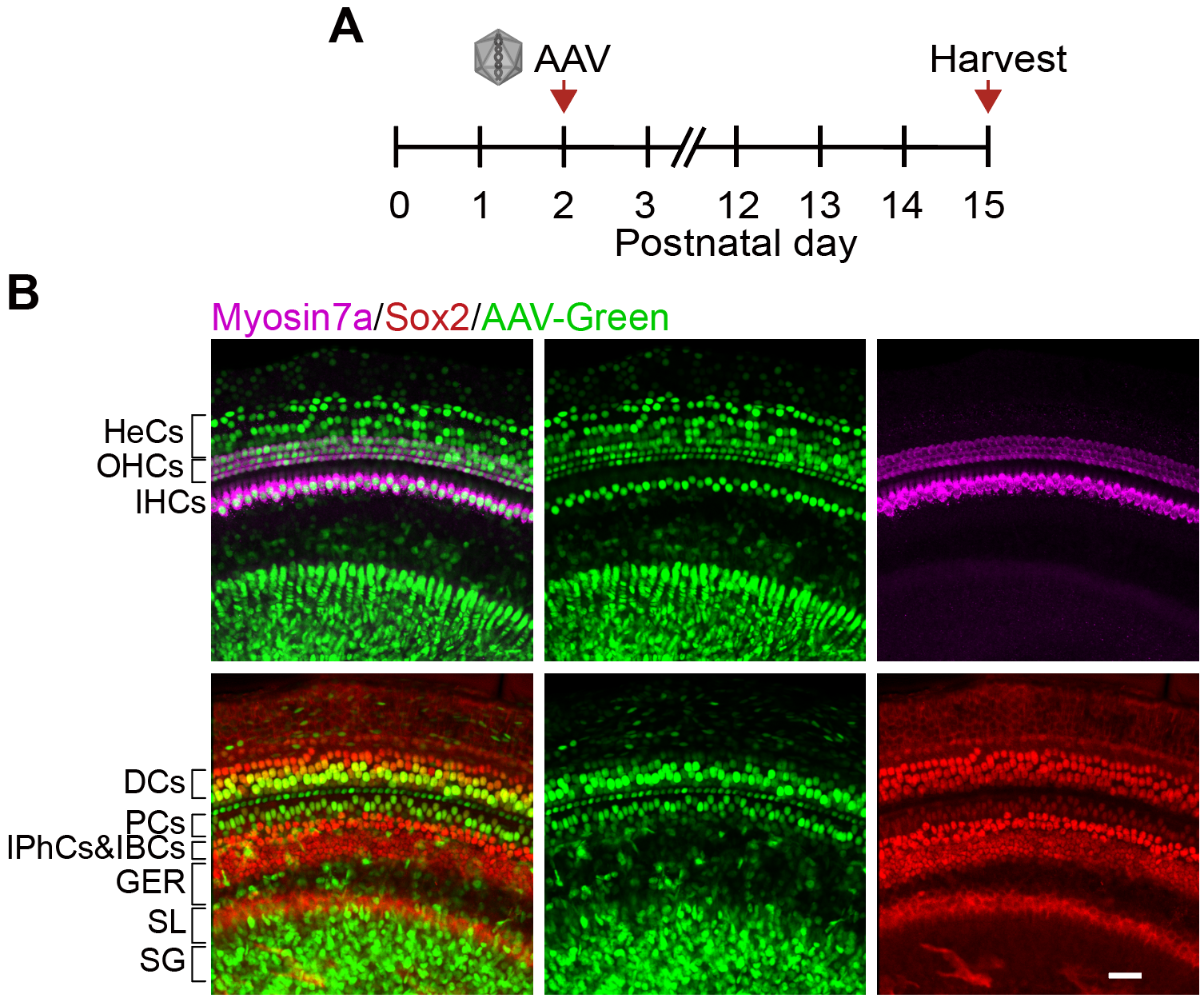


**Fig. S1.** **AAV-ie-NLS-mNeonGreen was highly effective at infecting HCs and SCs in mice.**

**(A)** Experimental design diagram. All the AAVs were injected to the mice left ear at a dose of 2.5E9 GCs.

**(B)** Confocal images of Myosin7a (magenta), Sox2 (red), and NLS-mNeonGreen fluorescence (green) signals in the apical turns of cochleae delivered with AAV-control (AAV-ie-NLS-mNeonGreen). Scale bar, 20 μm.


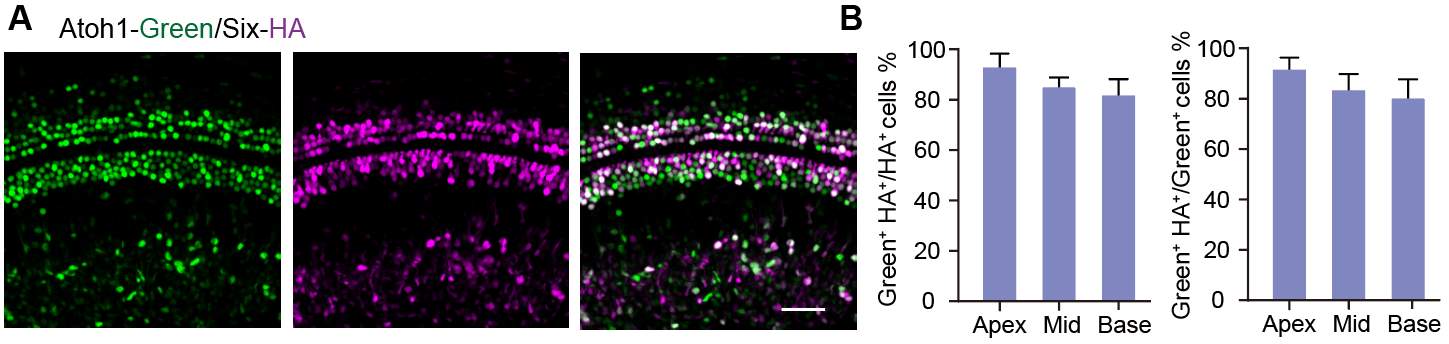


**Fig. S2. AAV-ie-mediated two AAVs can efficiently infect the same cells.**

**(A)** The immunofluorescence images of Atoh1-Green and Six1-HA signals in the apical turn of ear injected with AAV-ie-Atoh1-mNeonGreen-NLS and AAV-ie-Six1-HA mixture (1:1) at P1 mice. 2 weeks performed immunofluorescence staining, all the AAVs were delivered to the left ear, dose: 2.5E9 GCs per ear. Magenta: HA. Scale bar, 50 μm.

**(B)** The percentage of positive double Green^+^/HA^+^ cells in (A). N= 3, error bars are ±SEM.


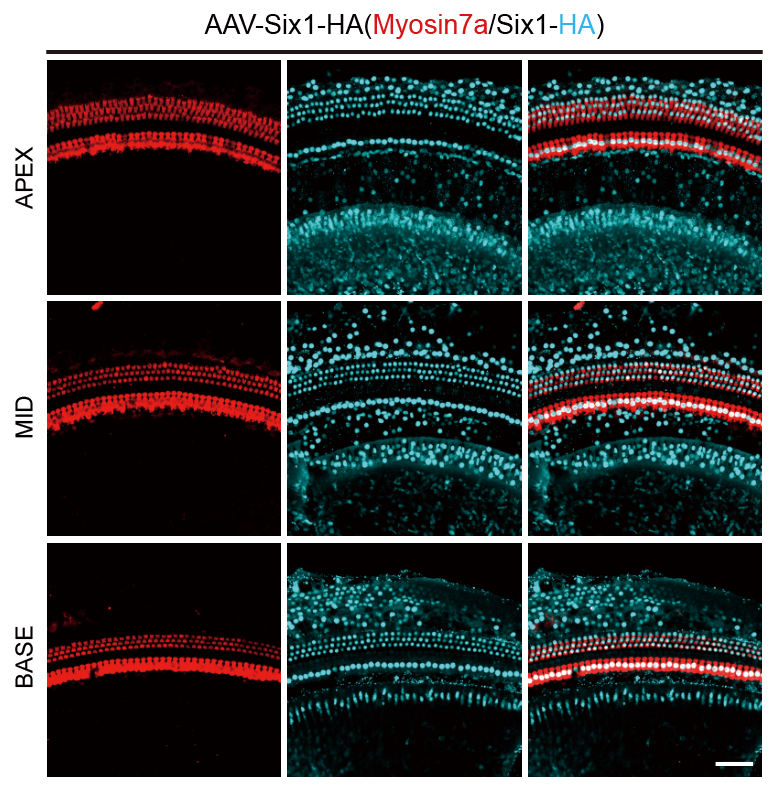


**Fig.** **S3. Forced Six1 expression in the cochlea was not capable of regenerating HCs.**

Representative confocal images of Myosin7a and Six1-HA signals in the basal, middle, and apical turns of left ear from P15 mice injected with AAV-*Six1* at a dose of 2.5E9 GCs at P2. Red: Myosin7a, cyan: Six1-HA. Scale bar, 50 μm.


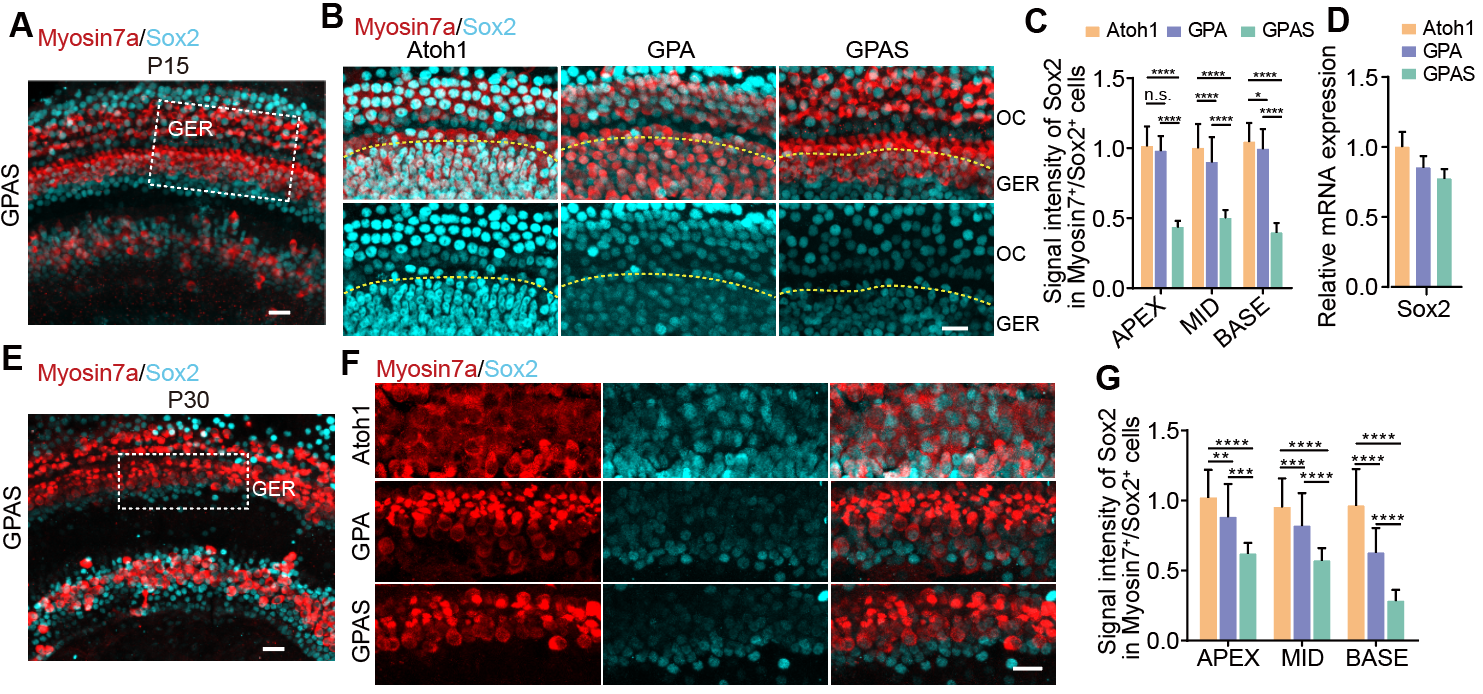


**Fig. S4.** **Sox2 staining in AAV-*Atoh1*, AAV-GPA, and AAV-GPAS-transduced mice.**

**(A)** The confocal images of Myosin7a (red) and Sox2 (cyan) signals in the apical turns of cochleae injected with AAV-GPAS in P15 mice. All the AAVs were injected to the mice left ear at a dose of 2.5E9 GCs. Scale bars, 20 μm.

**(B)** Representative confocal images of Myosin7a (red) and Sox2 (cyan) signals in the apical turns of left ear injected with AAV-*Atoh1*, AAV-GPA, and AAV-GPAS. The enlarge images and orthogonal views from (A) are shown. Scale bars, 20 μm.

**(C)** Signal intensity of Sox2 in Myosin7a ^+^/Sox2^+^ cells in ear transduced by AAV-Atoh1, AAV-GPA, and AAV-GPAS, corresponding to (B). N=4, error bars are ±SEM. **p <* 0.05, *****p <* 0.0001, n.s., no significance.

**(D)** Using qPCR analyzed the Sox2 mRNA expression in the left ear transduced with AAV-*Atoh1*, AAV-GPA, and AAV-GPAS. Raw results from 3 replicated experiments. Error bars are ±SEM.

**(E)** The immunofluorescence images of Myosin7a (red) and Sox2 (cyan) signals in the apical turns of P30 cochleae injected with AAV-GPAS at P2 at a dose of 2.5E9 GC in the left ear. Scale bars, 20 μm.

**(F)** The immunofluorescence images of Myosin7a (red) and Sox2 (cyan) signals in the apical turns of the left ear injected with AAV-*Atoh1*, AAV-GPA, and AAV-GPAS. The enlarge images and orthogonal views from (E) are shown. Scale bars, 20 μm.

**(G)** Signal intensity of Sox2 in Myosin7a^+^/Sox2^+^ cells in the left ear transduced by AAV-*Atoh1*, AAV-GPA, and AAV-GPAS, corresponding to (F). N=4, error bars are ±SEM. ***p <* 0.01, ****p <* 0.001, *****p <* 0.0001.


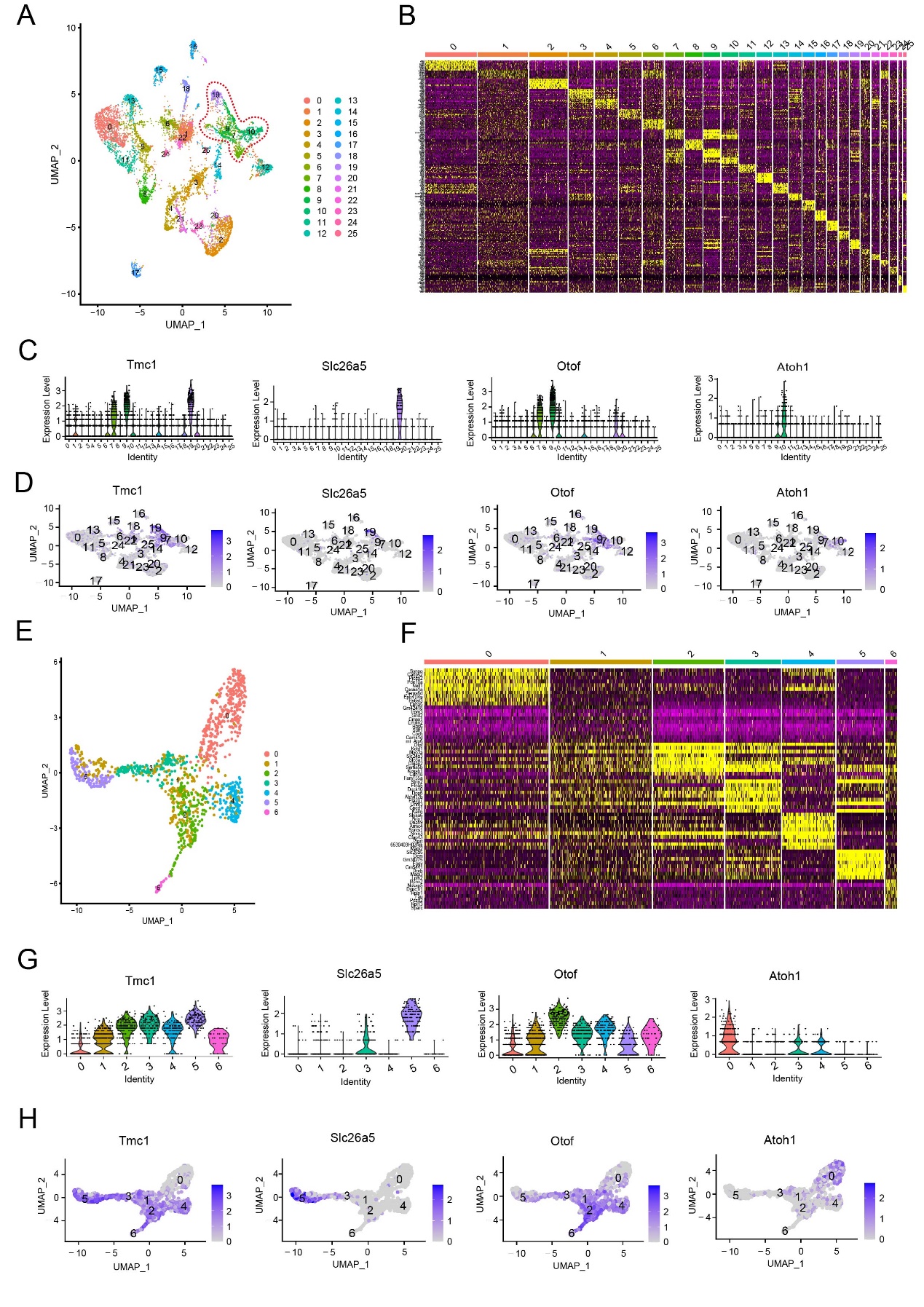


**Fig. S5.** **Single-nucleus transcriptomic profiles of cochlear epithelia with over-expression of *Atoh1*.**

**(A)** UMAP plot showing 26 unidentified clusters in the cochlear epithelium, with potential HC types outlined by the red dotted line.

**(B)** The heatmap revealing the top 10 differential expressed genes in every cluster related to (A).

**(C)** Violin plots highlighting well-known HC marker genes (*Tmc1*, *Slc26a5*, *Otof*, and *Atoh1*) that were distributed in *Seurat*-identified clusters and were highly enriched in clusters 7, 9, 10, and 19.

**(D)** Same as in (C), but presented as feature plots.

**(E)** UMAP plot showing seven clusters from the re-clustering of clusters 7, 9, 10, and 19 in (A).

**(F)** Heatmap plot of the top 10 differentially-expressed genes in different HC subtypes.

**(G)** Violin plots showing that *Tmc1*, *Slc26a5*, *Otof*, and *Atoh1* exhibited gradual or distinct patterns of gene expression in the seven subtypes in (E).

**(H)** Same as (G), but shown as feature plots of the sequential trajectory expression of *Tmc1*, *Slc26a5*, *Otof*, and *Atoh1*.


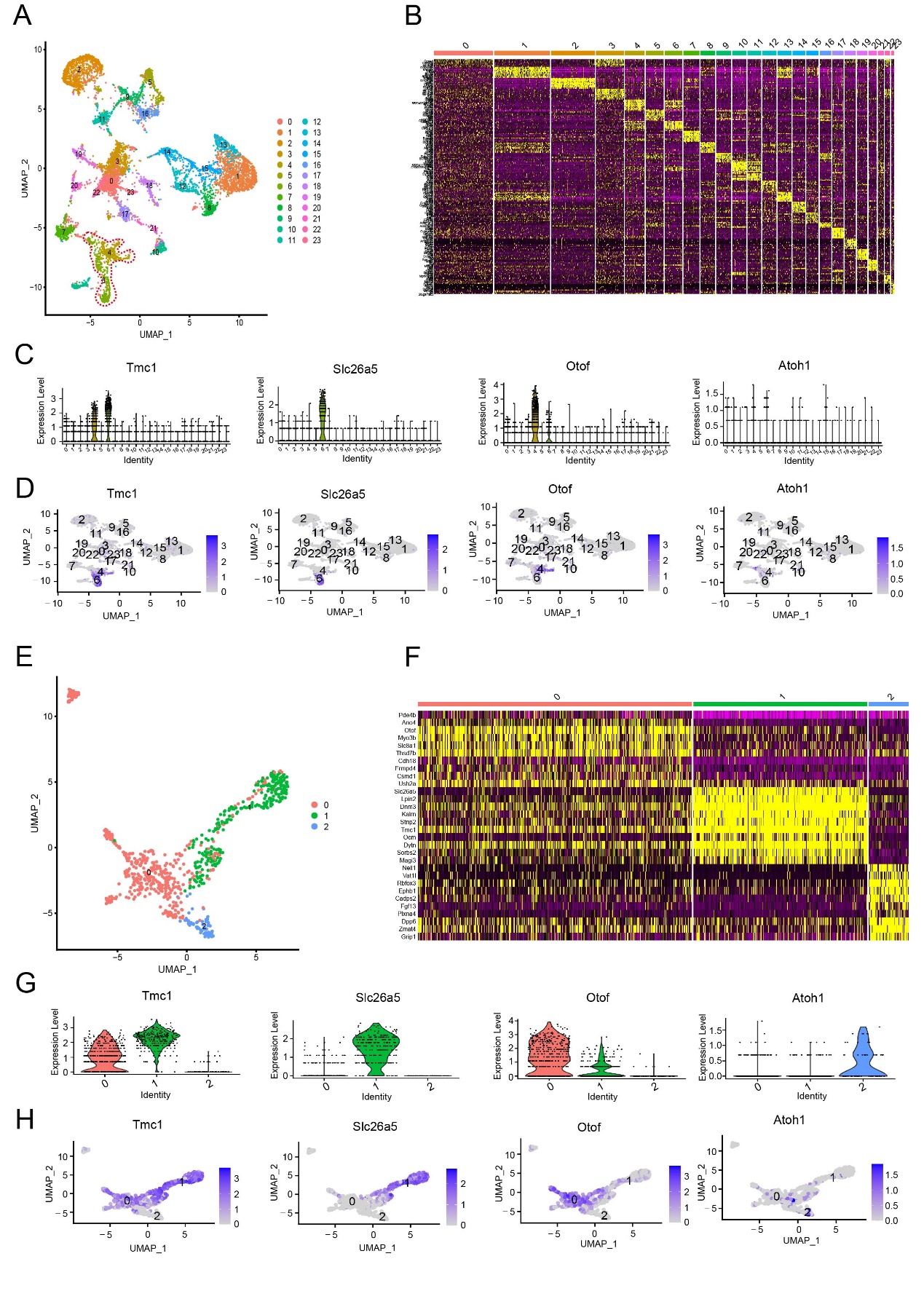


**Fig. S6. Single-nucleus transcriptome profiles of the cochlear epithelium with co-expression of *Gfi1*, *Pou4f3*, and *Atoh1*.**

**(A)** UMAP plot showing 24 clusters in the cochlear epithelium of the AAV-GPA group, with potential HC types outlined by the red dotted line.

**(B)** The heatmap revealing the top 10 differential expressed genes in every cluster related to (A).

**(C)** Violin plots highlighting well-known HC marker genes (*Tmc1*, *Slc26a5*, *Otof*, and *Atoh1*) that were distributed in *Seurat*-identified clusters and were highly enriched only in clusters 4 and 6.

**(D)** Same as in (C), but shown by feature plots.

**(E)** UMAP plot showing three clusters from sub-setting and re-clustering of cluster 4 and 6 in (A).

**(F)** Heatmap plotted of the top 10 differentially expressed genes in the HC subtypes.

**(G)** Violin plots showing *Tmc1*, *Slc26a5*, *Otof*, and *Atoh1* expression in the three subtypes in (E).

**(H)** Same as (G), but shown with feature plots.


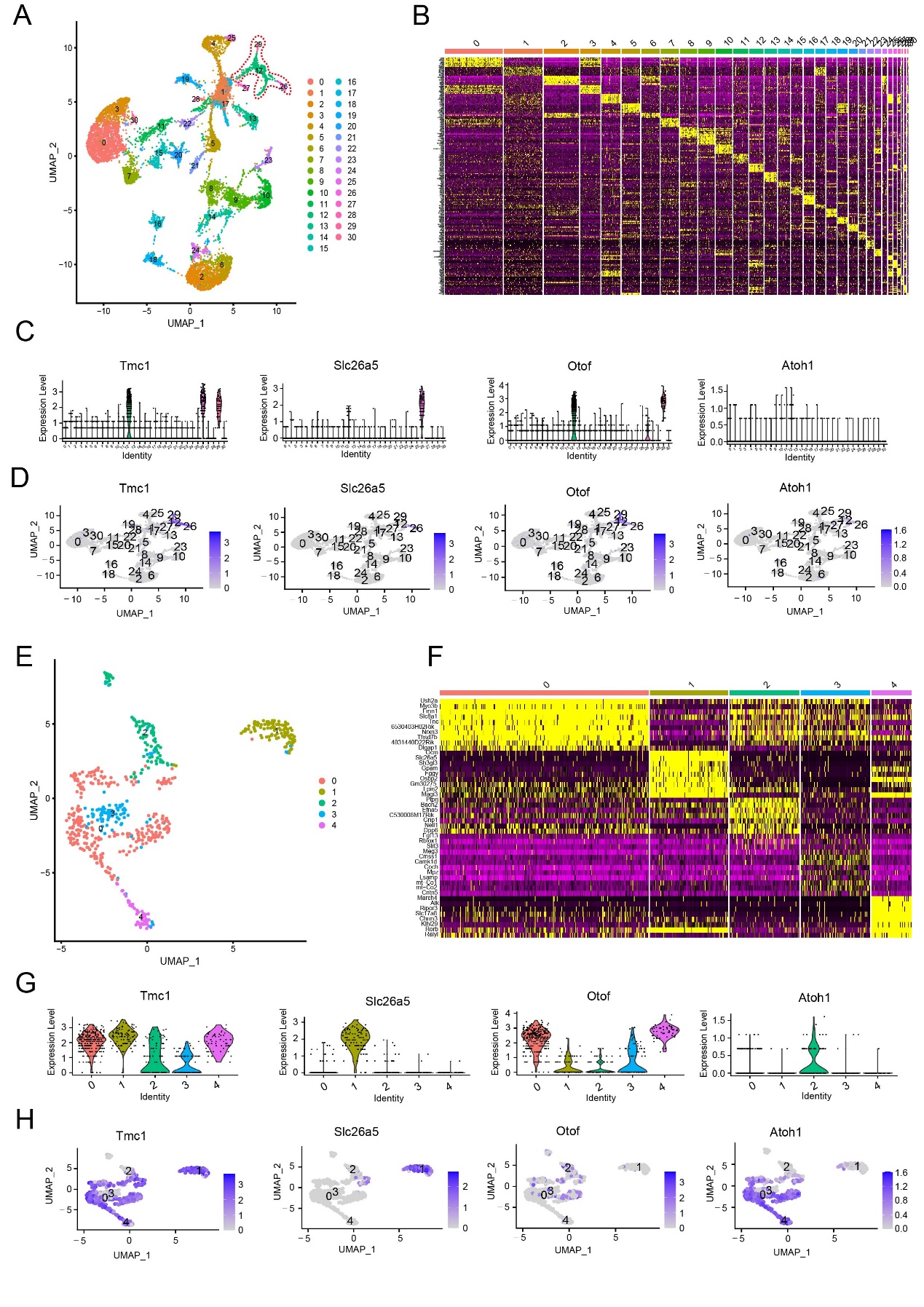


**Fig. S7. Single-nucleus transcriptome profiles of cochlear epithelium with co-expression of *Gfi1, Pou4f3, Atoh1*, and *Six1*.**

**(A)** UMAP plot showing 31 unnamed clusters in the cochlear epithelium of AAV-GPAS group, with potential HC types outlined by the red dotted line.

**(B)** The heatmap revealing the top 10 differential expressed genes in every cluster related to (A), with distance separation in each cluster.

**(C)** Violin plots showing the classical HC marker genes (*Tmc1*, *Slc26a5*, *Otof*, and *Atoh1*) that were highly enriched in clusters 12, 26, and 29.

**(D)** Same as in (C), but shown as feature plots.

**(E)** UMAP plot showing five clusters from re-clustering of clusters 12, 26, and 29 in (A).

**(F)** Heatmap plot of the top 10 differentially expressed genes of these HC subtypes.

**(G)** Violin plots showing that *Tmc1*, *Slc26a5*, *Otof*, and *Atoh1* exhibited gradual or distinct patterns of gene expression in the five subtypes in (E).

**(H)** Same as (G), but shown as feature plots illustrating the trajectory expression of *Tmc1*, *Slc26a5*, *Otof*, and *Atoh1*.


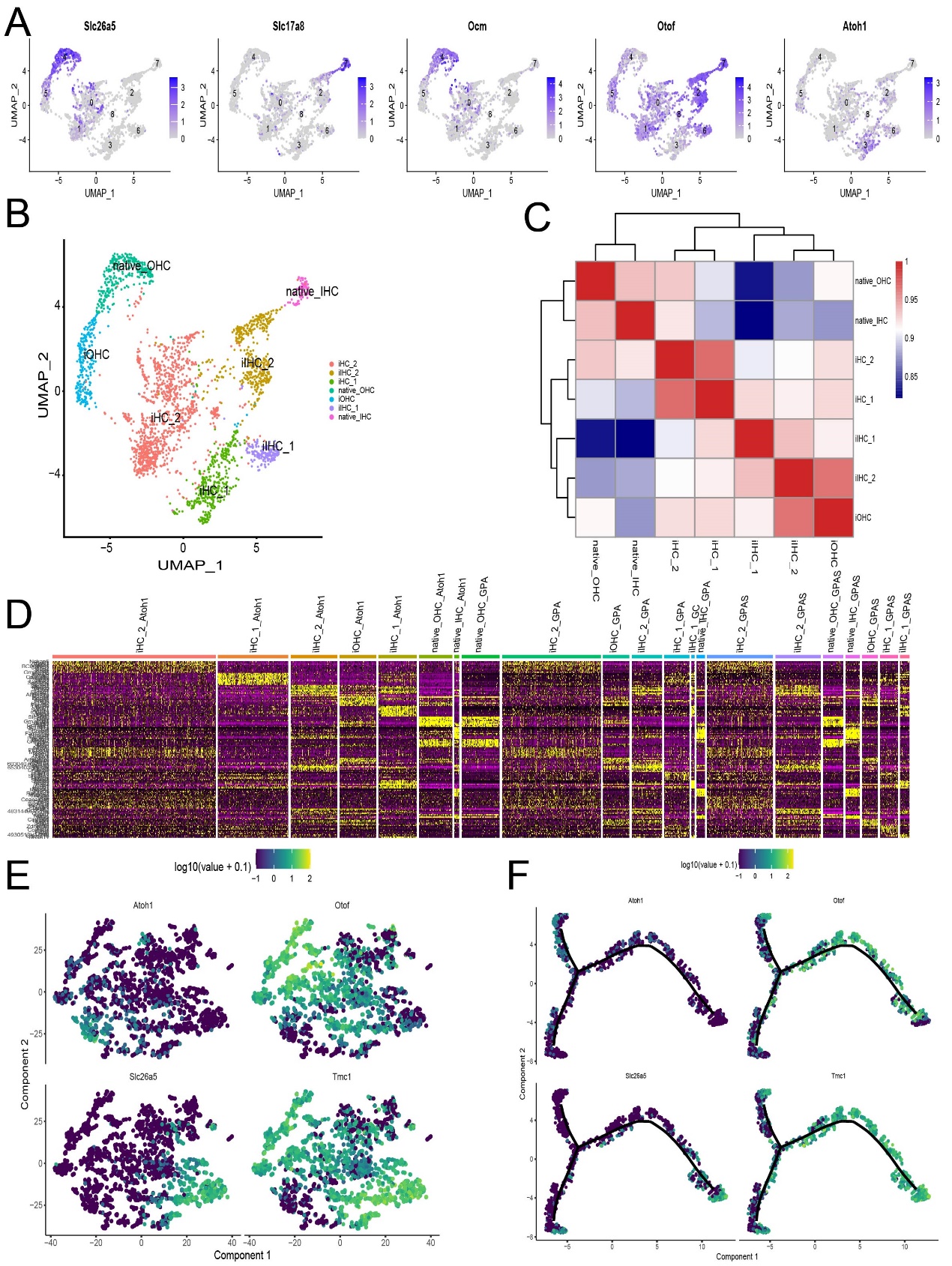


**Fig. S8. Single-nucleus transcriptome profiles of AAV-infected HCs with co-expression of one, three, or four transcription factors.**

**(A)** UMAP plots highlighting well-known HC marker genes (*Slc26a5*, *Slc17a8*, *Ocm*, *Otof*, and *Atoh1*) distributed in Seurat-identified clusters and highly enriched in different clusters.

**(B)** UMAP plot showing seven identified subtypes of AAV-infected HCs in the cochlear epithelium.

**(C)** Heatmap showing the correlation between different HC subtypes.

**(D)** The heatmap revealing the top 10 differential expressed genes in every cluster related to (B).

**(E)** Feature plots showing *Tmc1*, *Slc26a5*, *Otof*, and *Atoh1* expression in the seven subtypes in (B).

**(F)** Trajectory manifolds of HCs with well-known HC marker genes (*Slc26a5*, *Slc17a8*, *Ocm*, *Otof*, and *Atoh1*) for each respective cell fate.


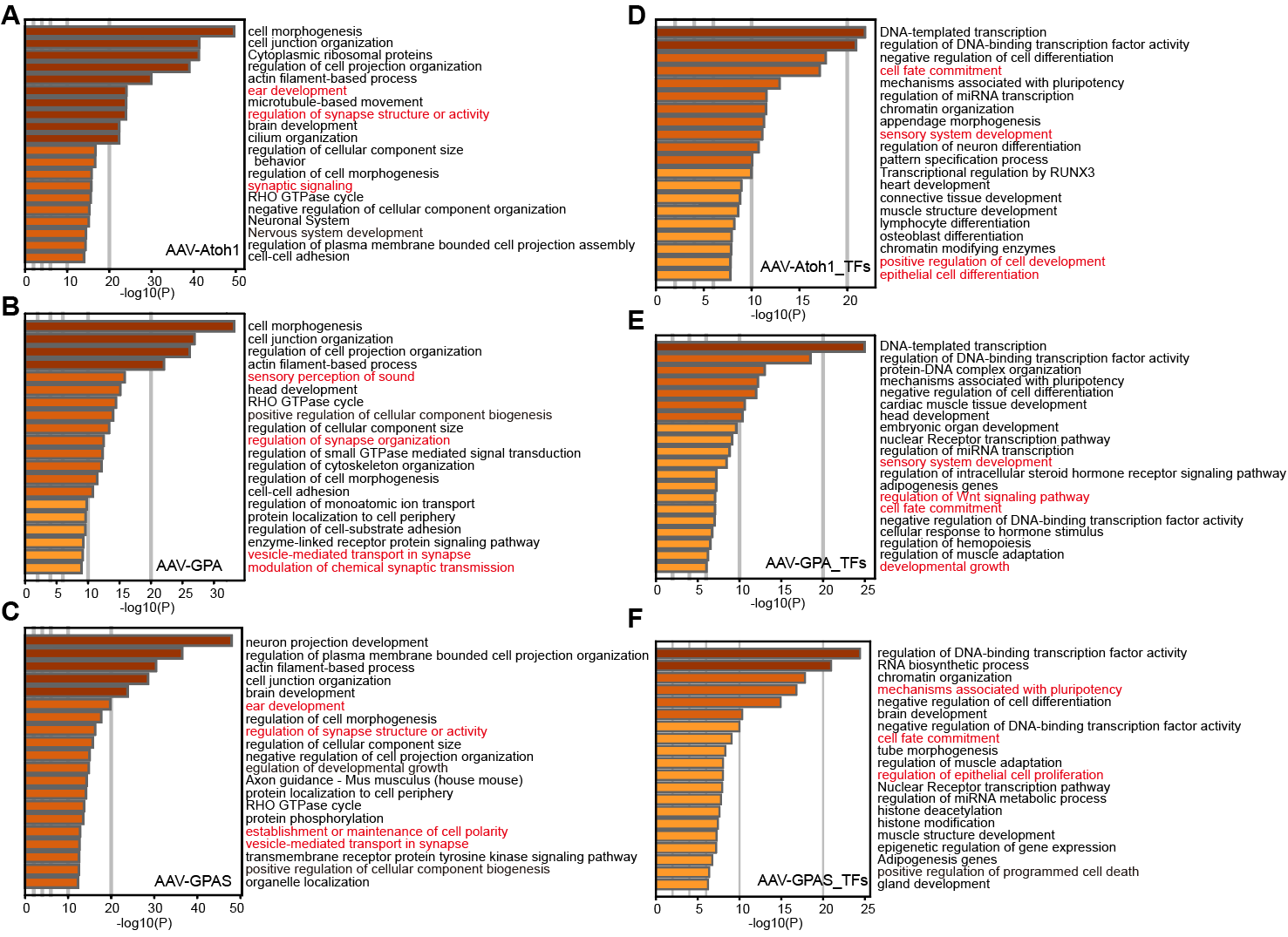


**Fig. S9. Go analysis of differential genes and transcription factors in the AAV-*Atoh1*, AAV-GPA, and AAV-GPAS groups.**

**(A-C):** Go analysis was performed on the differential genes of AAV-*Atoh1*, AAV-GPA, and AAV-GPAS groups using the Metascape website.

**(D-F):** Go analysis of transcription factors in the differential genes of AAV-*Atoh1*, AAV-GPA, and AAV-GPAS group was performed using the Metascape website.


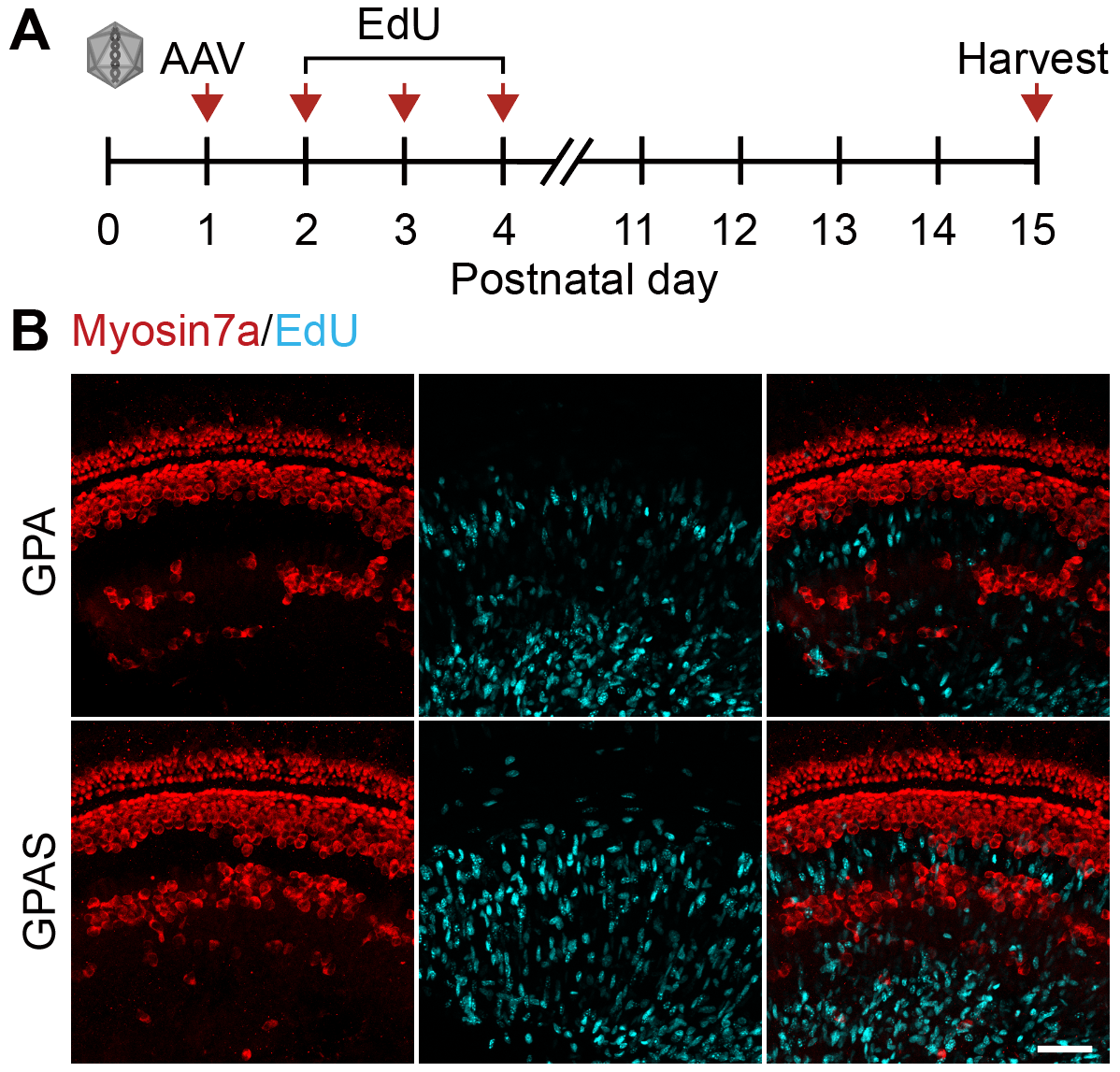


**Fig. S10.** **EdU staining in AAV-GPA and AAV-GPAS-transduced cochleae.**

**(A)** Experimental design diagram. All the AAVs were injected to the mice left ear at a dose of 2.5E9 GCs.

**(B)** The immunofluorescence confocal images of Myosin7a (red) and EdU (cyan) signals in the apical turns of the left ear injected with AAV-GPA/GPAS. Scale bar, 50 μm.


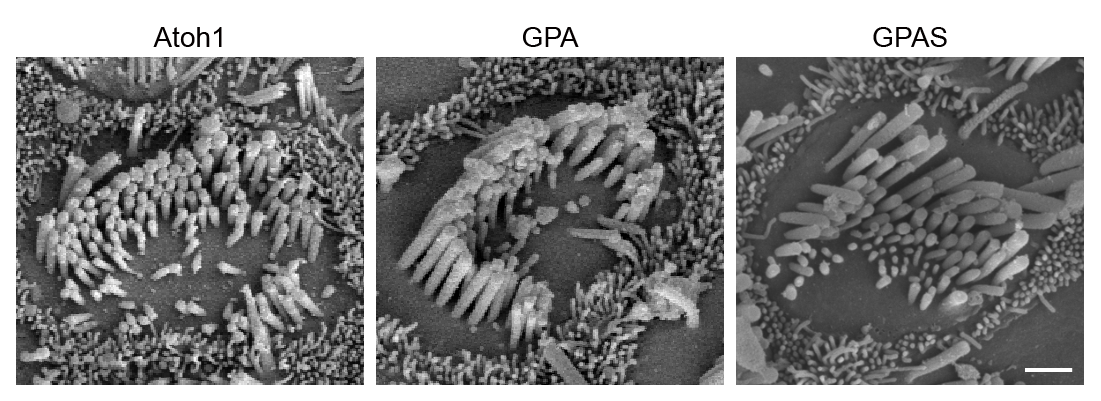


**Fig. S****11. Scanning electron microscope images of regenerated HCs.**

Representative [scanning electron microscope](https://www.sciencedirect.com/topics/neuroscience/scanning-electron-microscope) images of regenerated HCs in cochleae injected with AAV-*Atoh1*, AAV-GPA, and AAV-GPAS, respectively, Scale bars, 1 μm.


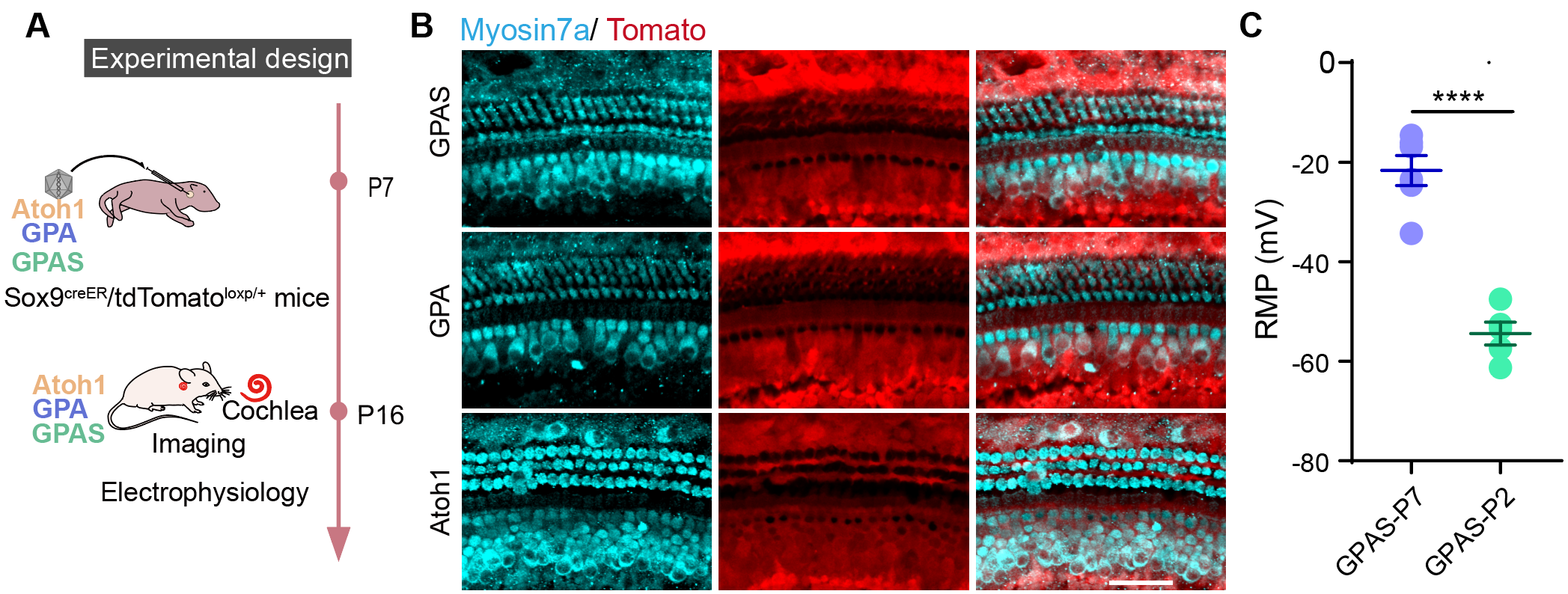


**Fig. S12. Regenerated HC source and maturity of P7 mice after AAV-GPAS injection.**

**(A)** Experimental design diagram. All the AAVs were delivered to the P7 mice’s left ear, dose: 2.5E9 GCs per ear.

**(B)** The immunofluorescence images of Myosin7a and Tomato signals in the apical turn of the ear injected with AAV-*Atoh1*, AAV-GPA and AAV-GPAS, respectively at P16 mice. Cyan: Myosin7a. Red: Tomato. Scale bar, 40 μm.

**(C)** Dot-plot showing the RMP recorded in HC-like cells from AAV-GPAS injected P2 and P7 mice. N=5, error bars are ±SEM. *****p <* 0.0001.


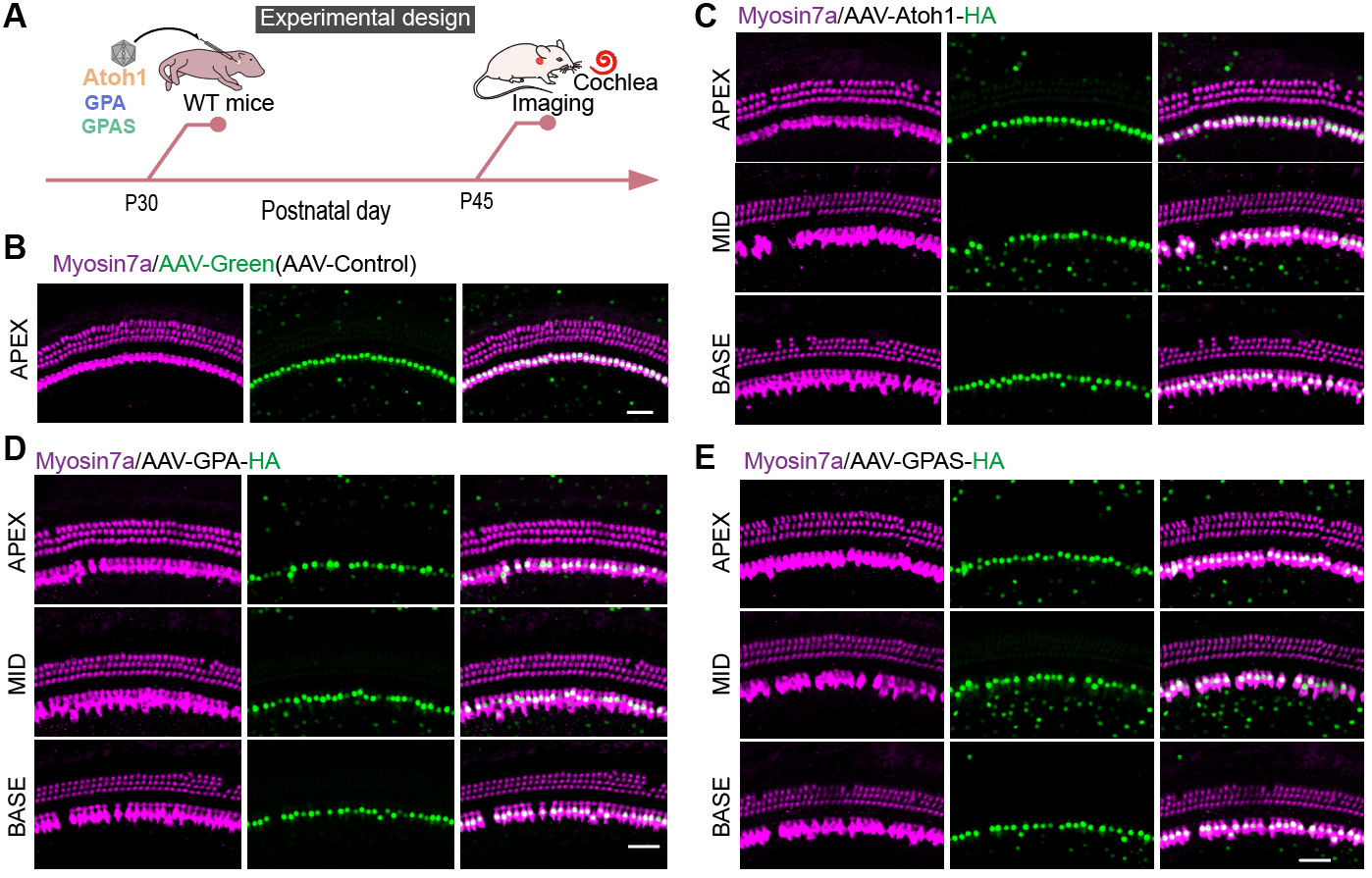


**Fig. S13. AAV-ie-mediated Atoh1, GPA, and GPAS incapably regenerated HCs in P30 mice.**

**(A)** Experimental design diagram. All the AAVs were delivered to the P30 mice’s left ear, dose: 2.5E9 GCs per ear.

**(B)** The immunofluorescence images of Myosin7a signals in the apical turn of the ear injected with AAV-control at P30 mice. Magenta: Myosin7a. Green: AAV-control. Scale bar, 40 μm.

**(C-E)** Representative confocal images of Myosin7a and HA signals in the apical to basal turns of the ear injected with AAV-*Atoh1*, AAV-GPA and AAV-GPAS respectively at P30 mice. Magenta: Myosin7a, Green: HA. Scale bar, 40 μm.


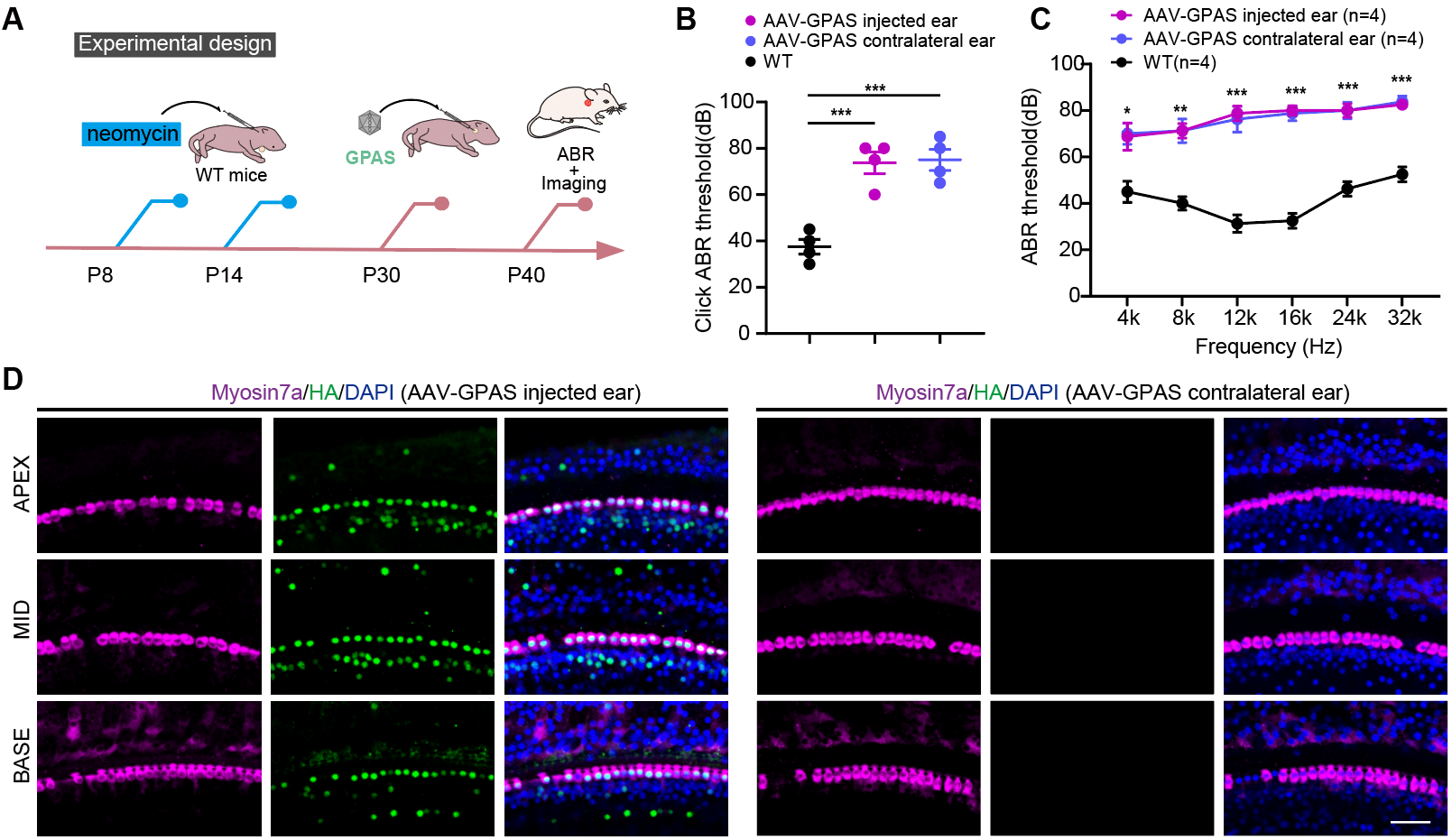


**Fig. S14: AAV-GPAS injection failed to restore hearing and HC regeneration in neomycin damaged model.**

**(A)** Experimental design diagram. The neomycin was injected into the P8-P14 mice at the dose of 200mg/kg, and the AAV-ie-GPAS was delivered to the P30 mice’s left ear, dose: 2.5E9 GCs per ear.

**(B)** The click ABR results of WT, AAV-GPAS injected ear and contralateral ear at P40 mice. N=4, error bars are ±SEM. ****p <* 0.001.

**(C)** The ABR results of WT mice, AAV-GPAS injected ear and contralateral ear at P40 mice. N=4, error bars are ±SEM. **p <* 0.005, ***p <* 0.01, ****p <* 0.001.

**(D)** The immunofluorescence images of Myosin7a and HA signals in the AAV-GPAS injected ear and contralateral ear. Magenta: Myosin7a, Green: HA. Scale bar, 50 μm.

**Table S1. Primers used in this manuscript.**

| Gene | Forward (5’-3’) | Reverse (5’-3’) |
| --- | --- | --- |
| Gfi1 | GTTCGAGGACTTTTGGAGGC | CTTGAAAGGCAGCGTGTAGG |
| Pou4f3 | ATTCTCCAGCCTACACTCCG | ATGATTCTTGCCGTGGGAGA |
| Atoh1 | GGGTGAGCTGGTAAGGAGAA | ACTACAACCCCACCCTTCAG |
| Six1 | CGAGGCCAAGGAAGGGAG | ACTCCTCTTCTGAGCTGGACATG |
| Gapdh | GGAGCCAAACGGGTCATCAT | TCACGCCACATCTTTCCAGA |
| WPRE | GTCAGGCAACGTGGCGTGGTGTG | GGCGATGAGTTCCGCCGTGGC |
| Chd7 | GTGAAGCTGTGTTGAAAGGCA | GTGAAGCTGTGTTGAAAGGCA |
| Ankrd6 | GTCGCTGCGCTTTCAGAAC | CTCGGCAAAGCTCCTCTTCTG |
| Supervillin | CAGGGCTAGTGACTCATCGG | GTCTGTGGCACGCATGTATC |
| Zeb1 | GCTGGCAAGACAACGTGAAAG | GCCTCAGGATAAATGACGGC |
| tdTomato-mutant | GGCATTAAAGCAGCGTATCC | CTGTTCCTGTACGGCATGG |
| tdTomato-wildtype | AAGGGAGCTGCAGTGGAGTA | CCGAAAATCTGTGGGAAGTC |
| Lgr5-wildtype | ATACCCCATCCCTTTTGAGC | GAACTTCAGGGTCAGCTTGC |
| Lgr5-mutant | CTGCTCTCTGCTCCCAGTCT | GAACTTCAGGGTCAGCTTGC |
| Sox9-wildtype | CTAGGCCACAGAATTGAAAGATCT | GTAGGTGGAAATTCTAGCATCATCC |
| Sox9-mutant | GCG GTC TGG CAG TAA AAA CTA TC | GTG AAA CAG CAT TGC TGT CAC TT |
